# Antioxidant activity and laxative effects of tannin-enriched extract of *Ecklonia cava* in loperamide-induced constipation of SD rats

**DOI:** 10.1371/journal.pone.0246363

**Published:** 2021-02-24

**Authors:** Ji Eun Kim, Yun Ju Choi, Su Jin Lee, Jeong Eun Gong, Young Ju Lee, Ji Eun Sung, Young Suk Jung, Hee Seob Lee, Jin Tae Hong, Dae Youn Hwang

**Affiliations:** 1 Department of Biomaterials Science (BK21 FOUR Program), College of Natural Resources & Life Science/Life and Industry Convergence Research Institute/Laboratory Animal Resources Center, Pusan National University, Miryang, Korea; 2 College of Pharmacy, Pusan National University, Busan, Korea; 3 Department of Food Science and Nutrition, College of Human Ecology, Pusan National University, Busan, Korea; 4 College of Pharmacy, Chungbuk National University, Chungju, Korea; Jadavpur University, INDIA

## Abstract

To investigate the role of tannin-enriched extracts of *Ecklonia cava* (TEE) on the regulation of oxidative balance and laxative activity in chronic constipation, we investigated alterations after exposure to TEE, on constipation phenotypes, muscarinic cholinergic regulation, and oxidative stress responses in the transverse colons of SD rats with loperamide (Lop)-induced constipation. This extract contains high levels of total condensed tannin content (326.5 mg/g), and exhibited high inhibitory activity against 2,2-diphenyl-1-picrylhydrazyl (DPPH) radicals. TEE treatment induced significant improvements in reactive oxygen species (ROS) production, superoxide dismutase (SOD) expression and nuclear factor erythroid 2-related factor 2 (Nrf2) phosphorylation in primary smooth muscles of rat intestine cells (pRISMCs) and transverse colon of constipation model. Also, Lop+TEE treated groups showed alleviated outcomes for the following: most stool parameters, gastrointestinal transit, and intestine length were remarkably recovered; a similar recovery pattern was observed in the histopathological structure, mucin secretion, water channel expression and gastrointestinal hormones secretion in the transverse colon; expressions of muscarinic acetylcholine receptors M2/M3 (mAChR M2/M3) and their mediators on muscarinic cholinergic regulation were significantly recovered. Taken together, these results provide the first evidence that TEE stimulates oxidative stress modulation and muscarinic cholinergic regulation when exerting its laxative effects in chronic constipation models.

## Introduction

Oxidative stress is the outcome due to an imbalance between the production of ROS and intracellular anti-oxidant defenses in the body, resulting in serious damage to various cells and tissues, as well as inducing numerous chronic diseases such as cancer, diabetes and inflammation [1]. This stress especially plays an important role in the pathogenesis of some chronic diseases associated with constipation [2]. Children with chronic constipation present with oxidative stress potential as well as decreased levels of vitamin C, vitamin E, SOD activity and catalase activity [3]. Also, oxidative stress (such as decreased SOD content and increased malondialdehyde (MDA) content) was observed in diphenoxylate-induced constipation SD rats [4]. A similar response was observed in Lop-induced constipation rats. Lop treated groups showed significantly decreased activity of three antioxidant enzymes (SOD, catalase (CAT) and glutathione peroxidase (GPx)), and increased levels of MDA and H_2_O_2_ [5]. Based on previous evidence correlating oxidative stress and constipation, anti-oxidants acting as free radical scavengers have been considered as a new strategy for constipation therapy through the prevention of oxidative reaction.

Only a few studies have reported the potential of natural products and functional foods with antioxidant activity can relieve symptoms of chronic constipation. Aqueous extract of *Malva sylvestris* L. remarkably reduced symptoms for Lop-induced constipation, including fecal number and weight, gastric emptying and gastrointestinal transit. Exposure to this extract also reduced the elevated MDA and H_2_O_2_ levels, and significantly enhanced the reduction of antioxidant enzymes (SOD, CAT and GPx) activity [5]. Moreover, the *Ficus carica* aqueous extract was reported to markedly reduce fecal markers, water content, oxidative stress indicators and lipid metabolism in Lop-induced constipation and dextran sulfate sodium (DDS)-induced acute colitis models [6]. However, evidence correlating antioxidants and constipation is very limited, although the laxative effects of numerous natural products, including *Asparagus cochinchinensis*, *L*. *platyphylla*, and galla rhois, are well known [7–9]. Therefore, the potential for antioxidant activity and laxative effects of *E*. *cava*has received great attention since several extracts containing tannin, such as galla rhois, *Maeya micrantha*, *Senna macranthera* and *Urginea indica* Kunth, are known to improve symptoms of constipation in animal models [8, 10–12].

In the present study, in order to evaluate the potential as a new natural medicine, we undertook to investigate the role of TEE on oxidative stress regulation and the laxative effect in a Lop-induced constipation model. Our results provide the first scientific evidence that the tannin containing natural product, TEE, successfully induces the suppression of oxidative stress and laxative effects in the constipated animal model.

## Materials and methods

### Collection of *E*. *cava* samples and preparation of TEE

Dry samples of *E*. *cava* were obtained from Parajeju Com. (Jeju, Korea). Voucher specimens of *E*. *cava* (WPC-19-001) were deposited at the functional materials bank of the Wellbeing RIS Center, Pusan National University. Samples were also identified by Dr. Jung Youn Park at the Biotechnology Research Division, National Fisheries Research and Development Institute. Briefly, dried sample of *E*. *cava* was reduced to a powder using a pulverizer (MF-3100S, Hanil Electric Co., Seoul, Korea), after which a solution of TEE was obtained via extraction for 24 h at 50°C, using a fixed liquor ratio (ratio of solid powdered *E*. *cava* to dH_2_O solvent, 1:10) in a circulating extraction equipment (SHWB-30/45; Woori Science Instrument Co., Pocheon, Korea). This solution was then passed through a 0.4 μm filter and subsequently concentrated, after which the pellets of TEE were dried in a rotary evaporator (EYELA, Tokyo, Japan) and stored at -80°C until used.

### Determination of bioactive compounds in TEE

The total phenolic contents of TEE were determined using the Folin-Ciocalteu method, as previously described [13]. Briefly, a mixture of TEE solution (1 mL) and Folin-Ciocalteu reagent (5 mL; Sigma-Aldrich Co., St. Louis, MO, USA) was incubated at room temperature for 5 min. This mixture was subsequently added to 15 mL 20% Na_2_CO_3_ and vortexed for 30 sec, after which the absorbance was repeatedly measured at 765 nm using a VersaMax plate reader (Molecular Devices, Pasadena, CA, USA). A standard calibration curve was generated using different concentrations of gallic acid (Sigma-Aldrich Co.), and concentration of the total phenolic compounds in TEE was presented as the gallic acid equivalent (mg) of the extract. The total flavonoid contents in TEE were measured as previously described [14]. Briefly, 20 μL samples of varying concentrations of TEE were mixed with 60 μL 5% NaNO_2_ and 60 μL 10% AlCl_3_ (Sigma-Aldrich Co.). Following incubation at 25°C for 5 min, the absorbance was measured using a VersaMax plate reader (Molecular Devices). A standard calibration curve was created using different concentrations of catechin (Sigma-Aldrich Co.). The total flavonoid contents of the TEE are presented as catechin equivalents (mg) of the extract. Finally, the total condensed tannin content was determined using the Vanillin method [15]. The extract powder of TEE was collected after dissolving in 0.5 ml 80% methanol. This extract (100 μL) was mixed with 500 μL of mixture solution (1% vanillin/MeOH and 8% HCL/MeOH, 1:1 ratio), and then incubated at 30°C for 20 min. The absorbance was measured at 500 nm using a VersaMax plate reader (Molecular Devices). The total condensed tannin content was calculated from a calibration curve constructed using a purified (+)-catechin hydrate standard (Sigma-Aldrich Co.).

### Gas Chromatography-Mass Spectrometry (GC-MS) and Fourier transform infrared spectroscopy (FTIR) analyses

The identification of TEE components was performed by GC-MS (QP-2010A, Shimadzu, Japan) equipped with automatic thermal desorber (ATD 400, Perkin Elmer Ltd., Beaconsfield, UK). The capillary column AT-1 was 60 m × 0.32 mm × 1.0 μm and mass range was 20–350 m/z. The temperature ramp was programmed mode with an initial temperature of 35°C held for 10 min, from 35°C to 120°C with linear rate 8°C/ 10 min, from 120°C to 180°C with linear rate 12°C/min, from 180°C to 230°C with linear rate 15°C/min, and held this temperature for 10 min. Comparison of the experimental mass spectra with those stored in Wiley221, Nist21, Nist107 Library identified GC of fragmentation products of immature pine cone. Also, FTIR spectroscopy (Impact 400D, Nicolet, Madison, WI, USA) was used to confirm the components in TEE. For each IR spectrometer sample, 32 scans at a 4 cm^-1^ resolution between 400 and 4000 cm^-1^ was collected in transmittance mode.

### Free radical scavenging activity analysis

The scavenging activity of TEE against DPPH radicals was determined as described in a previous study [16]. Briefly, the pellet of TEE was dissolved in 50% ethanol (EtOH, 100 μL), and was further serially diluted into 12 concentrations (1 to 1,000 μg/mL). Each concentration of TEE was mixed with 100 μL of 0.1 mM DPPH (Sigma-Aldrich Co.) in 95% EtOH solution, or 100 μL of 95% EtOH solution (control), after which it was incubated for 30 min at room temperature. The absorbance of the reaction mixture was measured at 517 nm with a VersaMax plate reader (Molecular Devices). The scavenging activity of TEE for DPPH radical is expressed as the percent decrease in absorbance relative to the control. The half maximal inhibitory concentration (IC_50_) value is the substrate concentration that exerted 50% loss in the DPPH activity.

### Determination of intracellular ROS levels

To investigate the antioxidant effects of TEE, intracellular ROS levels were measured by staining with 2′,7′‑dichlorofluorescein diacetate (DCF‑DA; Sigma-Aldrich Co.). Briefly, pRISMCs were prepared using a method described in previous studies, with slight modification [17]. pRISMCs were seeded into 6-well plates and 96-well plates at a density of 3 × 10^4^ cells in 3 mL and 200 μL medium each, and incubated for 20–24 h at 37°C. After reaching 70–80% confluence, the wells containing cells were assigned to No, low concentration of TEE (LTEE), high concentration of TEE (HTEE) and Lop treated groups. LTEE and HTEE treated groups were treated with 200 μg/mL and 400 μg/mL of TEE, respectively, whereas the No treated group received the same volume of only 1x PBS. The Lop treated group was exposed to 20 μM of Lop for 12 h, and subsequently divided into a Lop+LTEE treated group and a Lop+HTEE treated group. The Lop+LTEE treated groups were administered 200 μg/mL of TEE, while the Lop+HTEE treated group received 400 μg/mL of TEE for 12 h. The treated cells were then incubated with 10 μM DCF‑DA for 30 min at 37°C, washed twice with 1x PBS, after which the green fluorescence was observed using EVOS™ M5000 Imaging System (Thermo Fisher Scientific Inc., Sunnyvale, CA, USA). The DCF-DA fluorescence intensity was measured with a SpectraMax iD5 (Molecular Devices) using 485 and 535 nm as excitation and emission filters respectively.

### Experimental design for animal study

The animal protocol to determine therapeutic effects of TEE was reviewed and approved by the Pusan National University-Institutional Animal Care and Use Committee (PNU-IACUC) based on the ethical procedures for scientific care (Approval Number PNU-2019-2458). All adult Sprague Dawley (SD) rats were purchased from Samtako BioKorea Inc. (Osan, Korea), and maintained at the Pusan National University-Laboratory Animal Resources Center, accredited by the Korea Food and Drug Administration (KFDA) (Accredited Unit Number-000231) and The Association for Assessment and Accreditation of Laboratory Animal Care (AAALAC) International (Accredited Unit Number; 001525). All animals were provided *ad libitum* access to a standard irradiated chow diet (Samtako BioKorea Inc.) and water. Throughout the experiment, rats were maintained in a specific pathogen-free (SPF) state under a strict light cycle (on at 08:00 h; off at 20:00 h) at 23±2°C and 50±10% relative humidity.

Laxative effects of TEE were measured as described in previous studies [8, 12]. Briefly, 8-week-old SD rats (male, 270 g, n = 28) were assigned to either a non-constipation group (No group, n = 7) or a constipation group (n = 21). Subcutaneous injection of Lop (4 mg/kg weight) in 0.5% Tween 20 in saline was administered twice daily (9 a.m. and 6 p.m.) for 3 days to induce constipation, whereas the non-constipation group received 0.5% Tween 20 in saline alone under the same pattern. At 9 a.m. on the 4^th^ day, the constipation group was divided into a Lop+Vehicle treated group (n = 7), Lop+LTEE treated group (n = 7), and Lop+HTEE treated group (n = 7). The Lop+LTEE treated group was orally administered a single dose of 200 mg/kg body weight TEE, while the Lop+HTEE treated group received a single dose of 400 mg/kg body weight TEE. The Lop+Vehicle treated group was administered the same volume of 1× PBS under the same pattern. At 9 a.m. on the 5^th^ day, total stools, urine, water and food were collected from the metabolic cage of each mice in subset group, and their levels were measured using appropriate methods. All SD rats were then euthanized using CO_2_ gas, after which transverse colon samples were acquired and stored at -70°C in Eppendorf tubes until assay (Fig 4A).

### Measurement of food intake and water consumption

The food weight and water volume of SD rats treated with Vehicle or TEE were measured at 9 a.m. on the 5^th^ day using an electrical balance (for food weight) and a measuring cylinder (for water volume). All measurements were performed two times to ensure accuracy. The average food intake and water consumption were then calculated using the measured data.

### Measurement of stool parameters and urine volume

Throughout the experimental period, SD rats were individually bred in metabolic cages to provide uncontaminated stool and urine samples (Daejong Instrument Industry Co., LTD, Seoul, Korea). Briefly, stools excreted from each SD rat were collected at 9 a.m. on the 5^th^ day, and weighed in duplicate using an electric balance. The total number of stools was counted two times per animal. The stool moisture content was also analyzed as follows:

Stoolmoisturecontent=(A‐B)/A×100

where, A is the weight of fresh stools collected after Lop administration, and B is the weight of stools after drying at 60°C for 24 h. Furthermore, urine volume collected at 9 a.m. on the 5^th^ day was measured two times per sample using a cylinder.

### Measurement of gastrointestinal transit ratio and intestinal length

The gastrointestinal transit ratio was measured by the method described previously [18]. Briefly, SD rats were fasted for 18 h prior to the experiment, but were allowed to consume water *ad libitum*. Each rat in the subset group was fed 2 mL of charcoal meal (3% suspension of activated charcoal in 0.5% aqueous methylcellulose) (Sigma-Aldrich Co.). After 30 min of treatment, the rat was euthanized using CO_2_, and the intestinal tract was collected from the abdominal cavity. Intestinal charcoal transit ratio was calculated as follows:

Charcoaltransitratio(%)=[(totalsmallintestinelength–transitdistanceofcharcoalmeal)/totalsmallintestinelength)]x100

The total intestinal length was also measured from stomach to anus in duplicate.

### Western blotting analysis

Total proteins were collected from pRISMCs and transverse colons of SD rats treated with Lop or TEE, using the Pro-Prep Protein Extraction Solution (Intron Biotechnology Inc., Seongnam, Korea), according to manufacturer’s protocol. The acquired proteins were subsequently centrifuged at 13,000 rpm at 4°C for 5 min, after which total protein concentrations were determined using a SMARTTM Bicinchoninic Acid Protein assay kit (Thermo Fisher Scientific Inc.). Proteins (30 μg) were subjected to 4–20% sodium dodecyl sulfate-polyacrylamide gel electrophoresis (SDS-PAGE) for 3 h, and the resolved proteins were transferred to nitrocellulose membranes for 2 h at 40 V. The membranes were then probed with the following primary antibodies, overnight at 4°C: anti-Gα (ab128900, 1:1,000, Abcam, Cambridge, UK), anti-mAChR M2 (AMR-002, 1:1,000, Alomone Labs, Jerusalem, Israel), anti-mAChR M3 (AMR-006, 1:1,000, Alomone Labs), anti-PKC (2058s, 1:1,000, Cell Signaling Technology Inc., Danvers, MA, USA), anti-p-PKC (9376s, 1:1,000, Cell Signaling Technology Inc.), anti-PI-3K (4292s, 1:1,000, Cell Signaling Technology Inc.), anti-p-PI3K (4228s, 1:1,000, Cell Signaling Technology Inc.), anti-SOD (ab13498, 1:1,000, Abcam), anti-Nrf2 (ab137550, 1:1,000, Abcam), anti-p-Nrf2 (PA5-67520, 1:1,000, Invitrogen Co., Carlsvad, CA, USA), or anti-actin (4967s, 1:3,000, Sigma-Aldrich Co.). Membranes were subsequently washed with washing buffer (137 mM NaCl, 2.7 mM KCl, 10 mM Na_2_HPO_4_, 2 mM KH_2_PO_4_, and 0.05% Tween 20), followed by incubation with 1:1,000 diluted horseradish peroxidase-conjugated goat anti-rabbit IgG (Zymed Laboratories, South San Francisco, CA, USA) for 2 h at room temperature, after which the blots were developed using a Chemiluminescence Reagent Plus kit (Pfizer Inc., Morris Plains, NJ, USA). Signal images of each protein were subsequently acquired using a digital camera (1.92 MP resolution) of the FluorChem^®^ FC2 Imaging system (Alpha Innotech Corporation, San Leandro, CA, USA). Protein densities were semi-quantified using the AlphaView Program, version 3.2.2 (Cell Biosciences Inc., Santa Clara, CA, USA).

### Quantitative real time–polymerase chain reaction (qRT-PCR) analysis

The frozen transverse colon tissue was chopped with scissors and homogenized in RNA Bee solution (Tet-Test Inc., Friendswood, TX, USA). Total RNA molecules were isolated by centrifugation at 15,000 rpm for 15 min, after which RNA concentration was measured by the Nano Drop Spectrophotometer (Allsheng, Hangzhou, China). To examine the expression of SOD gene, total RNA (5 μg) from transverse colon tissue was annealed with 500 ng of oligo-dT primer (Thermo Fisher Scientific Inc.) at 70°C for 10 min. Complementary DNA (cDNA) was synthesized using the Invitrogen Superscript II reverse transcriptase (Thermo Fisher Scientific Inc.). qPCR was performed with the cDNA template obtained (2 μL) and 2 × Power SYBR Green (6 μL; Toyobo Life Science, Osaka, Japan) containing specific primers as follows: SOD, sense primer: 5′-GTGAA CCAGT TGTGT TGTCA GGAC-3′, anti-sense primer: 5‘- GATGG AATGC TCTCC TGAGA GTGAG ATC-3’; MUC2, sense primer. 5’-GCTGC TCATT GAGAA GAACG ATGC-3’ and anti-sense primer. 5’-CTCTC CAGGT ACACC ATGTT ACCAG G-3’; AQP3, sense primer. 5’-GGTGG TCCTG GTCAT TGGAA-3’, anti-sense primer. 5’-TCAAC CCTGC CCGTG ACT-3’; AQP8, sense primer. 5’-GTAGT ATGGA CCTAC GTGAG ATCAA GG-3’, anti-sense primer. 5’-AGAAC CTTTC CTCTG GACTC ACCAC C-3’; β-actin, sense primers. 5’-TGGAA TCCTG TGGCA TCCAT GAAAC-3’, anti-sense primers. 5’-TAAAA CGCAG CTCAG TAACA GTCCG-3’, respectively. qPCR was performed for 40 cycles using the following sequence: denaturation at 95°C for 15 sec, followed by annealing and extension at 70°C for 60 sec. Fluorescence intensity was measured at the end of the extension phase of each cycle. Threshold value for fluorescence intensities of all samples was set manually. The reaction cycle at which the PCR products exceeded this fluorescence intensity threshold during the exponential phase of PCR amplification was considered as the threshold cycle (Ct). Expression of the target gene was quantified relative to that of the housekeeping gene β-actin, based on a comparison of the Cts at constant fluorescence intensity, as per the Livak and Schmittgen’s method [19].

### Histopathological analysis

Transverse colons collected from No, Lop+Vehicle, Lop+LTEE and Lop+HTEE treated SD rats were fixed in 10% formalin for 48 h. Samples were subsequently embedded in paraffin wax, after which they were cut into 4 μm thick sections and stained with hematoxylin and eosin (H&E, Sigma-Aldrich Co.). The sections were then analyzed by light microscopy for mucosal thickness, flat luminal surface thickness and number of goblet cells in transverse colons, applying the Leica Application Suite (Leica Microsystems, Glattbrugg, Switzerland).

Mucin staining was achieved by fixing the transverse colons collected from SD rats of all subset groups in 10% formalin for 48 h, then embedding the samples in paraffin wax and sectioning into 4 μm thick slices that were subsequently deparaffinized with xylene and rehydrated. The mounted tissue sections were rinsed with distilled water and stained using an Alcian Blue Stain kit (IHC WORLD LLC, Woodstock, MD, USA), and morphological features in the stained colon sections were observed by light microscopy. Furthermore, the histopathological analyses including slide preparation, H&E staining, mucin staining and microscopic observation were conducted randomly.

### Analysis of SOD activity

SOD activity in the transverse colon tissue was assessed by applying a calorimetric assay and reagents provided in the SOD assay kit (Dojindo Molecular Technologies Inc., Kumamoto, Japan). Initially, transverse colon tissues (100 mg) were homogenized in 600 μL of sucrose buffer (0.25M sucrose, 10 mM HEPES, 1 mM EDTA, pH 7.4) using a glass homogenizer. Total protein was harvested by centrifugation of the homogenate at 10,000× g for 60 min, then stored at −70°C until assayed. To determine the SOD activity level, the tissue lysate was diluted with the dilution buffer or saline to the following ratios: 1, 1/5, 1/52, 1/53, 1/54, 1/55, and 1/56. Aliquots of each lysate solution (25 μL) were subsequently placed in individual wells of a 96-well plate, along with 200 μL of the WST-1 working solution. In addition, 20 μL of the enzyme working solution was added to each well, followed by thorough mixing. The enzyme reaction was induced by incubating the prepared plate at 37°C for 20 min, after which the absorbance of each well was measured at 450 nm using a spectrophotometer. Finally, the level of SOD activity was directly calculated using the equation: SOD activity (inhibition rate %) = [(A blank 1−A blank 3)−(A sample−A blank 2)] /(A blank 1−A blank 3)×100, where, A blank 1, 2, and 3 indicate the absorbances of blanks 1, 2, and 3, respectively, and A sample is the absorbance of the sample.

### Analysis of CAT activity

CAT activity in the transverse colon was assessed using the EZ-Catalase assay kit (DoGenBio, Seoul, Korea). After preparation of transverse colon homogenates, a volume of 25 μL of tissue sample was mixed with 25 μL of H_2_O_2_ solution. Subsequently, Oxi-Probe/Horse radish peroxidase solution (100 μM and 0.4 Units/mL dissolved in 1 × reaction buffer, pH 7.5) was added to each well, and incubated at room temperature for 30 min in the dark. Activity was monitored by measuring the fluorescent intensity using a Glomax microplate reader (Promega, Madison, WI, USA). Results were corrected by protein concentration determined with the BCA protein assay (Thermo Fisher Scientific Inc.), and expressed as units per mg of brain protein. One unit of catalase was defined as 1 μmol H_2_O_2_ decomposed per min.

### Measurement of gastrointestinal (GI) hormone concentrations

The concentration of cholecystokinin (CCK), gastrin and somatostatin (SS) were quantified using ELISA kits (Cusabio Biotech Co., Ltd., Wuhan, China), according to the manufacturer’s instructions. Briefly, transverse colon tissues (100 mg) were homogenized in ice-cold 1× PBS (pH 7.2–7.4) using a glass homogenizer (Sigma-Aldrich Co.). Resultant tissue lysates were then centrifuged at 1,000 × g for 5 min at 4°C, after which the supernatant was collected for analysis. After addition of the three specific hormone antibodies (separately in each well), the supernatant was incubated for 1 h at 37°C, to which HRP-Streptavidin solution was subsequently added and further incubated for 1 h at 37°C. This was followed by addition of the TMP One-Step Substrate Reagent, followed by incubation of the mixture for 30 min at 37°C. The reaction was terminated following addition of the stop solution. Finally, absorbance of the reaction mixture was read at 450 nm using the Molecular Devices VersaMax Plate Reader (Molecular Devices).

### Statistical analysis

Statistical significance was evaluated using the One-way Analysis of Variance (ANOVA) (SPSS for Windows, Release 10.10, Standard Version, Chicago, IL, USA), followed by Tukey post hoc t-test for multiple comparison. All values are expressed as the means ± SD, and a p-value (p < 0.05) is considered statistically significant.

## Results

### Bioactive components of TEE

**Fig 1:**
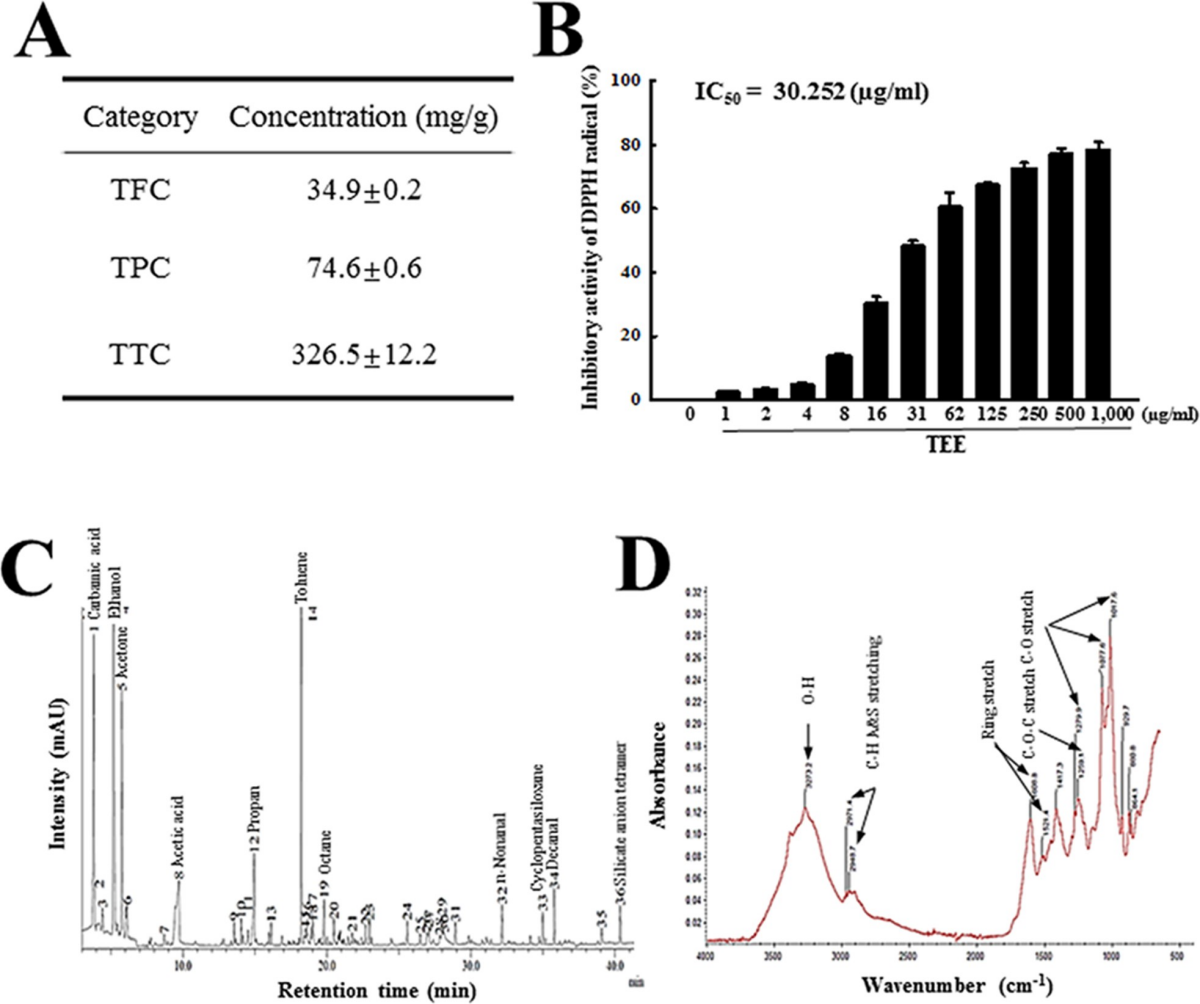
Phytochemical composition and component conformation in TEE. (A) Total flavonoids, phenols and condensed tannin content were analyzed in mixtures containing different concentrations of TEE. (B) Free radical scavenging activity of TEE. DPPH radical scavenging activity was assayed in a mixture containing 0.1 mM DPPH and varied concentrations of TEE (1–1,000 μg/mL). (C) GC-MS spectra of TEE. Major peak in GC/MC chromatogram indicated the name of component. (D) FTIR spectrum of TEE. The functional group of organic compound indicated into major peak. Three samples were assayed in duplicate by DPPH analysis. The data are reported as the mean ± SD. *, p < 0.05 compared with the No treated group. #, p < 0.05 compared with the Lop+Vehicle treated group. Abbreviations: DPPH, 2,2-diphenyl-1-picrylhydrazyl radical; IC_50_, half maximum inhibitory concentration.

We first analyzed the phytochemical composition of TEE to evaluate antioxidant activity of this extract. As presented in Fig 1A, total condensed tannin was detected at high levels (326.5 mg/g) as compared to other bioactive components. Total phenolic and total flavonoid contents were determined to be 74.6 mg/g and 34.9 mg/g, respectively. Also, the inhibitory activity of TEE against DPPH radical was dose-dependently and significantly increased, with an IC_50_ value of 30.25 μg/mL (Fig 1B). Furthermore, the components in TEE were identified using GC-MS and FTIR method. The major components of TEE were founded to be carbamic acid, ethanol, acetone, toluene, decanal, silicate anion tetramer, n-nonanal (Fig 1C). FTIR spectra showed the stretching vibrations of O–H groups in alcohols (3274 cm^-1^), C-H antisym and sym stretching vibration in hemicellulose, lignin, cellulose and tannin (2971 and 2948 cm^-1^), aromatic ring vibration (CĽC stretching) in lignin and tannin (1608 and 1521 cm^-1^), syringyl/guaicyl ring breathing in lignin (1279 cm^-1^), C–O–C stretching in lignin (1259 cm^-1^) and C–O–C pyranose ring skeletal vibration in hemicellulose and cellulose (1077 and 1017 cm^-1^) (Fig 1D). These results indicate that TEE is a tannin-enriched extract with potentially high antioxidant activity.

### Anti-oxidative activity of TEE in pRISMCs

**Fig 2:**
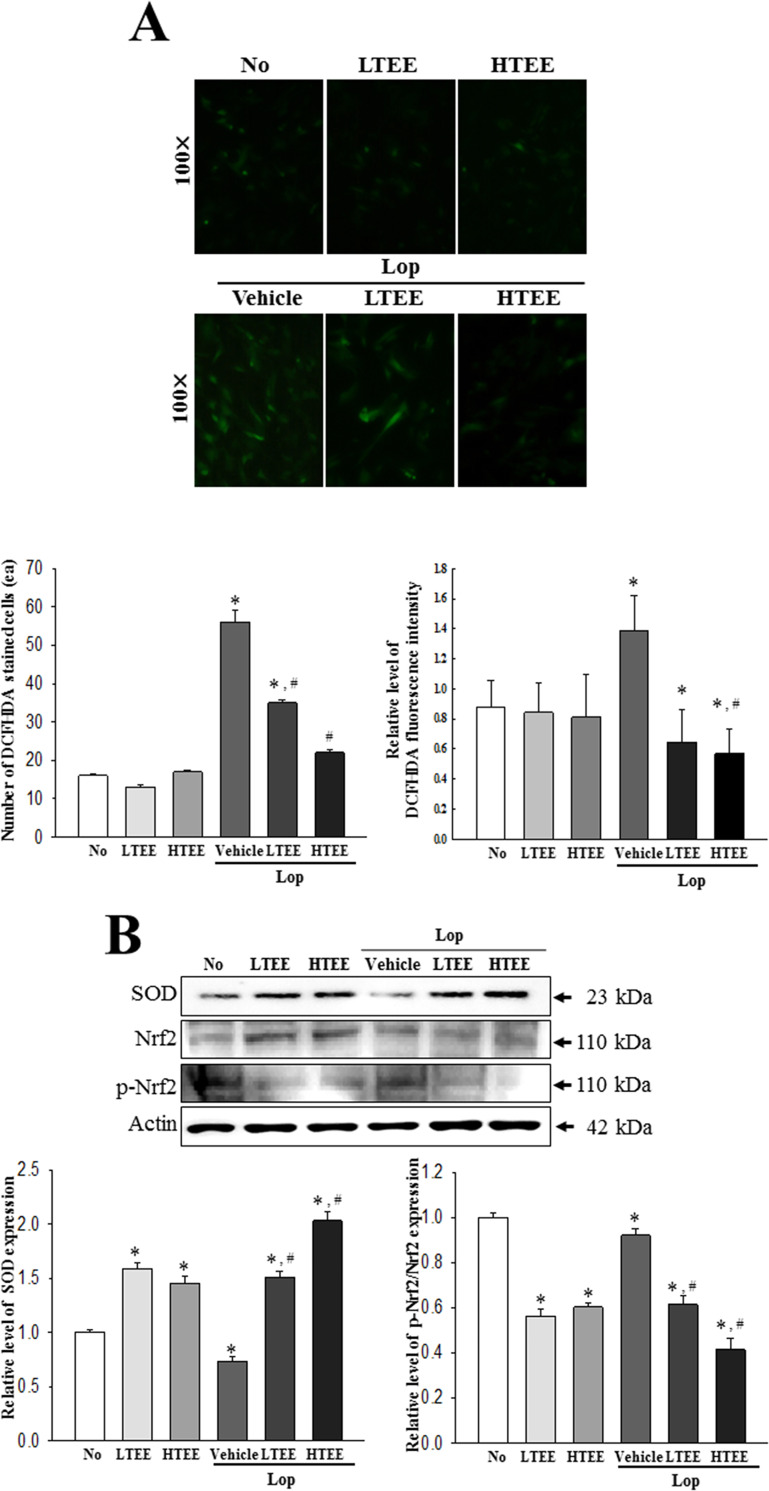
Antioxidant activity of TEE *in vitro*. (A) Determination of intracellular ROS production. After DCF-DA treatment into pRISMCs of each subset group, green fluorescence-stained cells were observed and counted using a fluorescent microscope, at 200× magnification. The fluorescence intensity was measured by ELISA reader. (B) Detection of SOD, Nrf2 and p-Nrf2 protein. Total cell lysates were prepared from pRISMCs treated with TEE or Lop+TEE, as described in Materials and Methods. The expression level of three proteins were detected with specific antibodies and quantified using an imaging densitometer. Three samples were assayed in duplicate by DCF-DA staining assay and western blotting. The data are reported as the mean ± SD. *, p < 0.05 compared with the No treated group. #, p < 0.05 compared with the Lop+Vehicle treated group. Abbreviations: ROS, Reactive oxygen species; DCF-DA, 2’,7’-dichlorofluorescein diacetate; SOD, Superoxide dismutase; pRISMCs, primary smooth muscle of rat intestine cells.

To examine the antioxidant activity of TEE in primary cells derived from colon of rats, alterations in the levels of intracellular ROS production, SOD expression, and phosphorylation of transcription factor for antioxidant enzyme were measured in pRISMCs after exposure to Lop and TEE. Antioxidant activity of TEE was similar to the inhibitory effects against Lop-induced ROS production in pRISMCs, wherein a decreased number of DCF-DH stained cells and level of fluorescence intensity were obtained in the Lop+TEE treated groups (Fig 2A). Furthermore, the antioxidant responses of TEE were reflected in SOD expression and Nrf2-mediated transcriptional induction, wherein enhanced SOD expression and decreased phosphorylation of Nrf2 was observed in the Lop+TEE treated groups (Fig 2B). These results demonstrate that TEE stimulates the antioxidant defense in intestinal cells, which is likely to be related to the antioxidant activity observed in rat intestines.

### Antioxidant activity of TEE in transverse colon of Lop-induced constipation rats

**Fig 3:**
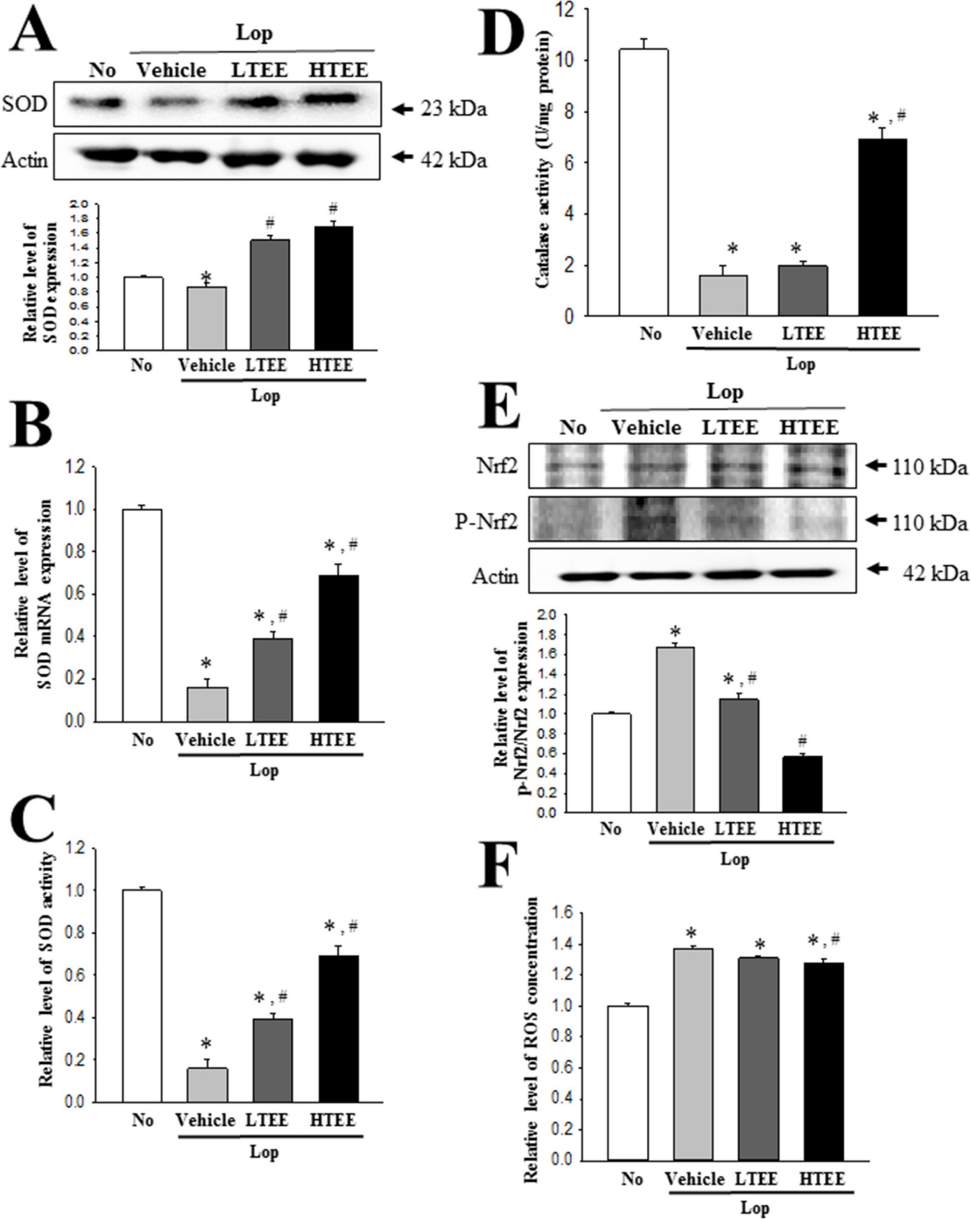
Antioxidant activity of TEE in transverse colon of Lop+TEE treated constipation rats. (A) Detection of SOD proteins. The expression level of SOD was measured by Western blot analysis using specific antibodies. The relative level of this protein in each group was calculated based on the intensity of actin, after calculating the intensity of each band. (B) Determination of SOD activity. The SOD activity level was measured in homogenates of transverse colon tissue collected from each subset group, as described in Materials and Methods. One SOD unit is defined as the amount of enzyme in 20 μL of the sample solution that inhibits the reduction reaction of water-soluble tetrazolium salt-1 (WST-1) with superoxide anion by 50%. (C) Detection of SOD mRNA. The levels of SOD transcripts in the total messenger RNA (mRNA) of brain were measured by RT-qPCR analyses using specific primers. The mRNA level of SOD gene was calculated, based on the intensity of actin as an endogenous control. (D) Detection of Nrf2, and p-Nrf2 protein. The expression level of the two proteins was measured by Western blot analysis using specific antibodies. Subsequently, the phosphorylation level of a specific protein was calculated by dividing the level of phosphorylated proteins by the level of total proteins. (E) Determination of CAT activity. The CAT activity was measured in homogenates of transverse colon collected from each subset group, as described in Materials and Methods. Catalase 1 unit is defined as the amount of enzyme required to decompose 1 μmole of H_2_O_2_ per min at pH 7.0 and 25°C. (F) Measurement of ROS concentration. ROS level was measured in homogenates of transverse colon collected from each subset group, as described in Materials and Methods. This assay kit has a detection sensitivity limit of 10 pM for DCF, and 40 nM for H_2_O_2_. Four to six rats per group were used in the preparation of total tissue homogenate and total RNA; Western blot analyses, ELISA, RT-PCR analysis were performed in duplicate for each sample. The data are reported as the mean ± SD. *, p<0.05 compared to the No treated group. ^#^, p<0.05 compared to the Lop+Vehicle treated group. Abbreviations: Lop, Loperamide; SOD, Superoxide dismutase; RT-qPCR, Quantitative real time-PCR; CAT, Catalase; ROS, Reactive oxygen species; DCF, 2’,7’-dichlorofluorescein; ELISA, Enzyme-linked immune sorbent assay.

To investigate whether the antioxidant activity of TEE in pRISMCs can be completely reflected into the regulation of oxidative defense in transverse colon of constipation rats, we measured alterations in the mRNA, protein and activity level of SOD, Nrf2 expression, CAT activity, and ROS level in the transverse colon of the Lop-induced constipation model after TEE treatment. Decreased levels of SOD mRNA, protein and activity in the Lop+Vehicle treated group were significantly increased after TEE administration, although the ratio of increase was varied. These alterations were reflected in the regulation of Nrf2 transcription factor involved in the regulation of antioxidant enzyme (Fig 3A–3C). The phosphorylation level of Nrf2 proteins was completely recovered in the Lop+LTEE and Lop+HTEE treated groups (Fig 3D). A similar recovery pattern was observed for CAT activity and ROS concentration in the transverse colon. Decreased level of CAT activity in the Lop+Vehicle treated group was enhanced in Lop+HTEE treated groups, whereas the increased ROS expression was significantly decreased in the same groups (Fig 3E and 3F). Taken together, these results indicate that that the antioxidant activity of TEE observed in primary cells derived from intestine may successfully exhibit in the transverse colon of animal model for constipation. Also, these data indicate that TEE with high antioxidant activity may associate with the regulation of transvers colon function including the defecation in the animal model for constipation.

### Effect of TEE administration on the feeding behavior and excretion parameters

**Fig 4:**
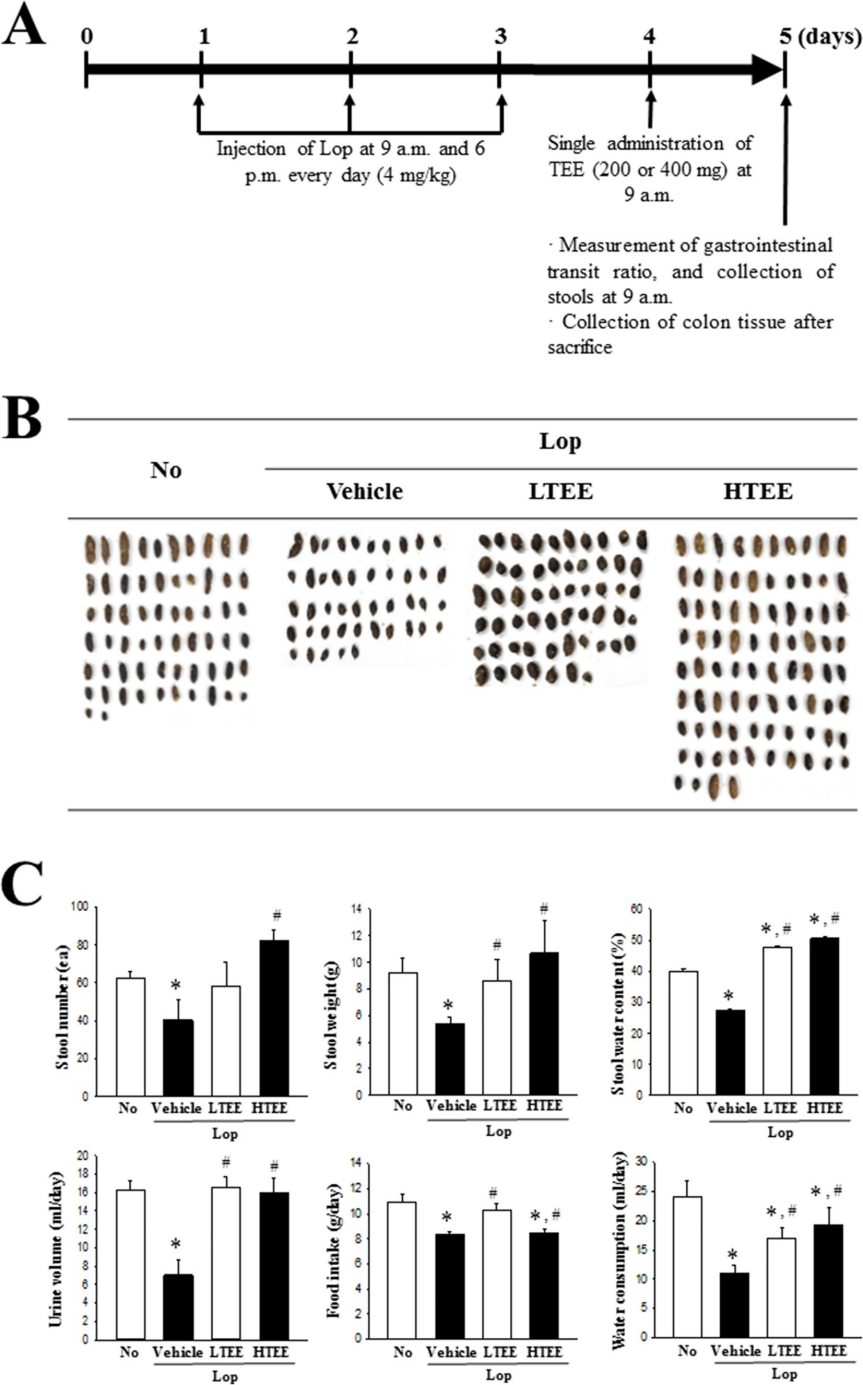
Experimental scheme, and stool parameters and feeding behavior analyses. (A) Schedule for constipation induction and TEE treatment. After three days of Lop injection, two different doses of TEE were singly administrated at 9 a.m. (B) Stool morphological characteristics. Digital camera images of stools were taken immediately after collection from the metabolic cage. (C) Total number and weight of stools were measured as described in Materials and Methods. Stool water content was calculated using the weight of fresh stools and dried weight. Food intake and water consumption was also calculated using the amount of feed (water) supplied and the amount of feed (water) remaining. Four to six rats per group were used for stool collection, and each parameter was assayed in duplicate. The data are reported as the mean ± SD. *, p < 0.05 compared with the No treated group. #, p < 0.05 compared with the Lop+Vehicle treated group. Abbreviation: Lop, Loperamide.

To investigate whether TEE administration stimulates defecation in constipated rats, we measured alterations in stool parameters, food intake and water consumption in Lop-induced constipated SD rats after single administration of TEE (Fig 4A). The stool parameters, including number, weight and water content, were significantly decreased by Lop treatment. However, these levels were almost recovered in the Lop+TEE treated groups, relative to the No and Lop+Vehicle treated groups. Also, above alterations of stool parameters were completely reflected in the stool morphology (Fig 4B). Recovery effects of TEE were also observed in urine volume and water consumption of Lop+TEE treated groups, while food intake was maintained constant in the same groups (Fig 4C). These results indicate that TEE stimulates defecation of Lop-induced constipation in SD rats, without significant alteration in their food intake.

### Effect of TEE administration on the gastrointestinal motility and intestinal length

**Fig 5:**
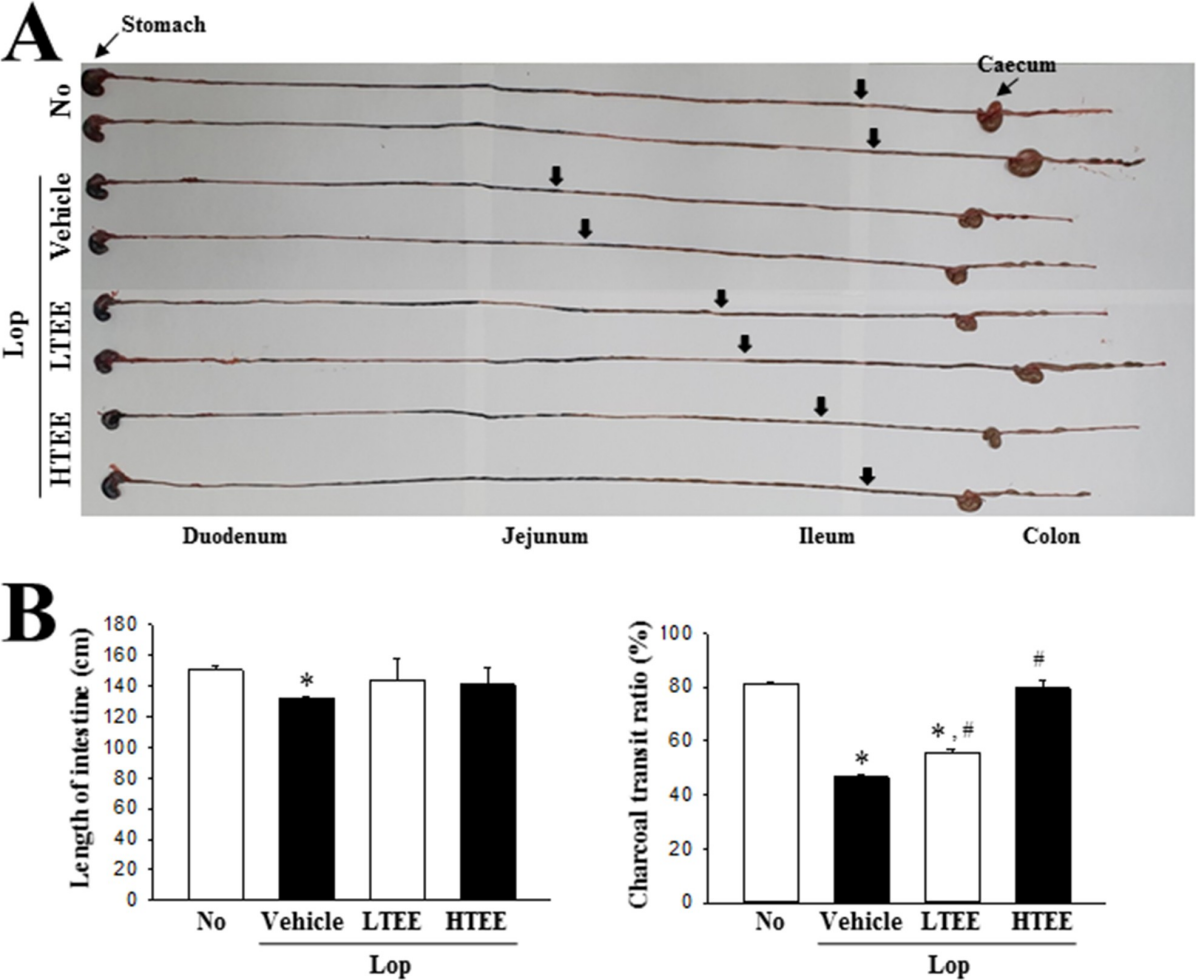
GI transit ratio and intestinal length in Lop+TEE treated constipation rats. (A) Actual image showing the charcoal meal transit and intestine. The total intestinal tract was excised from a rat of each subset group treated with charcoal meal powder. Morphology was observed using a digital camera. The arrows indicate position of the charcoal meal. (B) Transit ratio of the charcoal meal and the length of intestine. The total distance travelled by the charcoal meal from the pylorus was measured. The charcoal meal transit ratio was then calculated using total length of the intestine and distance of the charcoal meal. Four to six rats per group were used in the GI transit ratio test, and the charcoal meal transit distance and intestine length were measured in duplicate. The data are reported as the mean ± SD. *, p < 0.05 compared to the No treated group. #, p < 0.05 compared to the Lop + Vehicle-treated group. Abbreviations: GI, Gastrointestinal; Lop, Loperamide.

To investigate whether the defecation stimulation effects of TEE are accompanied by alterations in the gastrointestinal motility and intestinal length, we performed the charcoal meal transit test and intestine length analyses in Lop-induced constipation rats after TEE administration. The propulsion of charcoal meal was significantly decreased by 41.2% in the Lop+Vehicle treated group, as compared to the No treated group. However, these alterations showed dose-dependent recovery in the Lop+TEE treated groups. A similar recovery pattern was also detected for length of the intestine, although the range of transition was small (Fig 5). These results suggest that defecation stimulation effects of TEE are associated with regulation of the gastrointestinal motility and intestinal length in Lop-induced constipation rats.

### Effect of TEE administration on histopathological structure of the transverse colon

**Fig 6:**
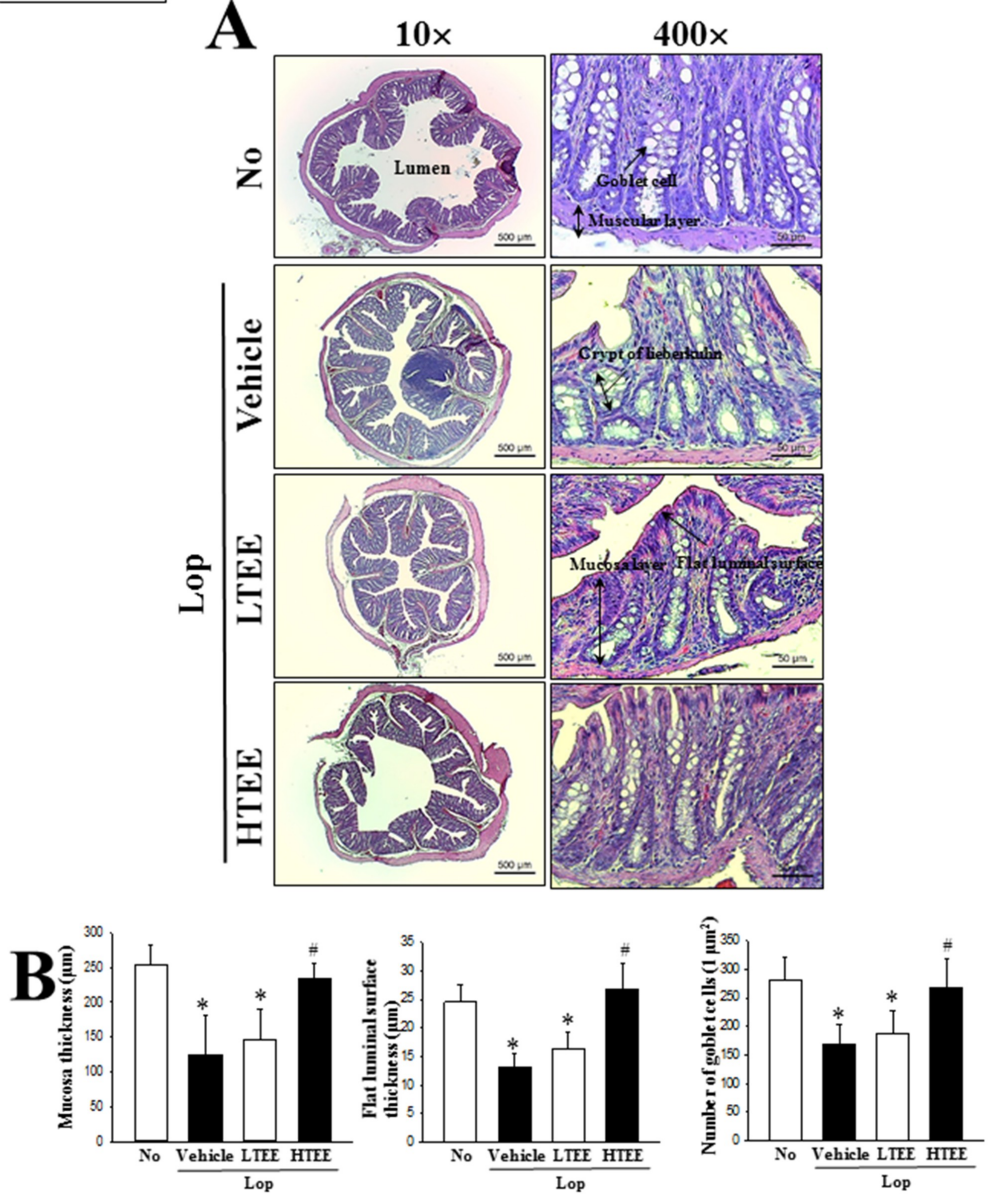
Histopathological structures of transverse colon in Lop+TEE treated constipation rats. (A) H&E stained sections of transverse colon from the No, Lop+Vehicle, Lop+LTEE or Lop+HTEE treated groups were observed at 40× (left column) and 400× (right column) using a light microscope. (B) Histopathological parameters were determined using the Leica Application Suite. Four to six rats per group were used in the preparation of H&E stained slide, and the histopathological parameters were measured in duplicate in each slide. The data are reported as the mean ± SD. *, p<0.05 compared to the No treated group. ^#^, p<0.05 compared to the Lop+Vehicle treated group. Abbreviations: H&E, Hematoxylin and eosin; Lop, Loperamide.

To investigate associated changes in the histological structure of the transverse colon due to the defecation stimulation effects of TEE in constipation rats, we measured alterations in the histological parameters indicating laxative effects in the H&E stained transverse colons of subset groups. Thickness of mucosa and flat luminal surface of transverse colon as well as width of the intestinal lumen were significantly decreased after Lop treatment, relative to the No treated group. However, these alterations were remarkably enhanced in Lop+LTEE and Lop+HTEE treated groups. Significant changes were also observed in the crypts of Lieberkuhn in transverse colon sections of Lop+TEE treated rats. The structure of crypts was remarkably altered from open form to the luminal face as well as recovery of irregular shape, distribution, and size of goblet cells in the crypts after TEE treatment (Fig 6). These findings show that defecation stimulation effects of TEE contribute to the recovery of structural abnormalities of the transverse colon of Lop-induced constipated SD rats.

### Effect of TEE administration on regulation of mucin secretion and water channel expression

**Fig 7:**
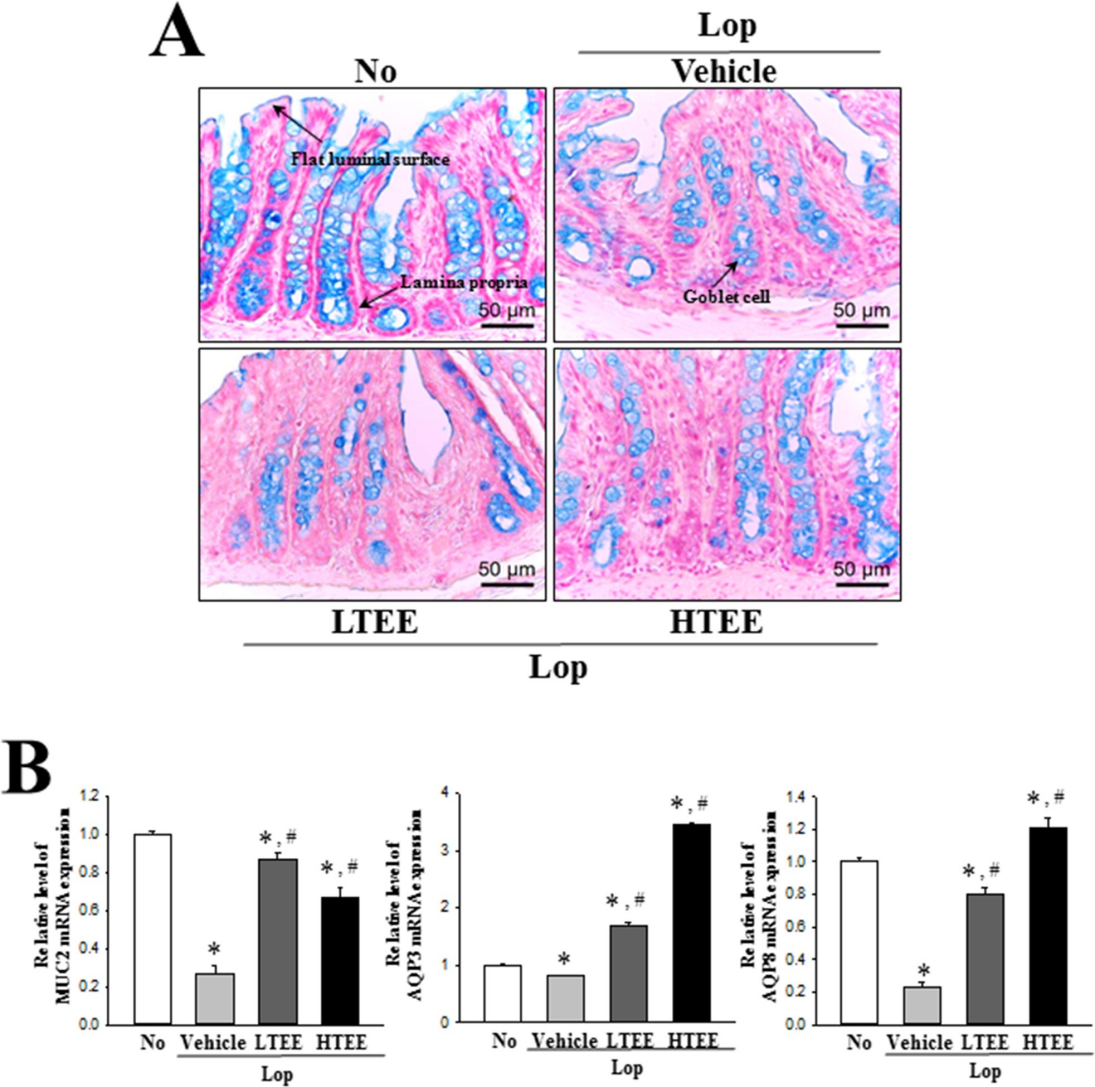
Mucin secretion and membrane water channel expression in Lop+TEE treated constipation rats. (A) Detection of mucin in Alcian blue stained transverse colon tissue. Mucin secreted from crypt layer cells was stained with Alcian blue at pH 2.5, and images were observed at 100× magnification. Four to six rats per group were used in the preparation of tissue slide, and Alcian blue staining analysis was performed in duplicate in each slide. (B) RT-qPCR analyses for MUC2, AQP3 and AQP8. The levels of MUC2, AQP3 and AQP8 transcripts in the total mRNA of transverse colons were measured by RT-qPCR using specific primers. The mRNA levels of three genes were calculated, based on the intensity of actin as an endogenous control. Four to six rats per group were used the preparation of total RNA, RT-qPCR analyses were assayed in duplicate for each sample. The data are reported as the mean ± SD. *, p<0.05 compared to the No treated group. ^#^, p<0.05 compared to the Lop+Vehicle treated group. Abbreviations: Lop, Loperamide; RT-qPCR, Quantitative real time-PCR; MUC2, Mucin 2; AQP, Aquaporin.

We next examined whether the defecation stimulation effects of TEE are accompanied by alterations in the regulation of mucin and water secretion in the transverse colon of constipation rats. To achieve these, changes in the levels of mucin secretion and regulatory protein expressions were measured in the transverse colon of SD rats after TEE treatment. Dark blue mucin staining in the goblet cells of crypt was regularly concentrated in the transverse colon of No treated groups, with decreasing intensity in the Lop+Vehicle treated group. However, TEE administration dramatically increased the staining in transverse colons of constipation rats (Fig 7A). These alterations were completely reflected in the level of MUC2 transcript obtained (Fig 7B). To correlate mucin secretion and water channel expression, we examined the levels of AQP3 and AQP8 mRNA, wherein decreased levels were obtained in the Lop+LTEE and Lop+HTEE treated groups (Fig 7B). These results show that the defecation stimulation effects of TEE are associated with enhanced ability to secrete mucin and expression of a membrane water channel in the transverse colon of Lop-induced constipated SD rats.

### Effect of TEE administration on regulation of GI hormones secretion

**Fig 8:**
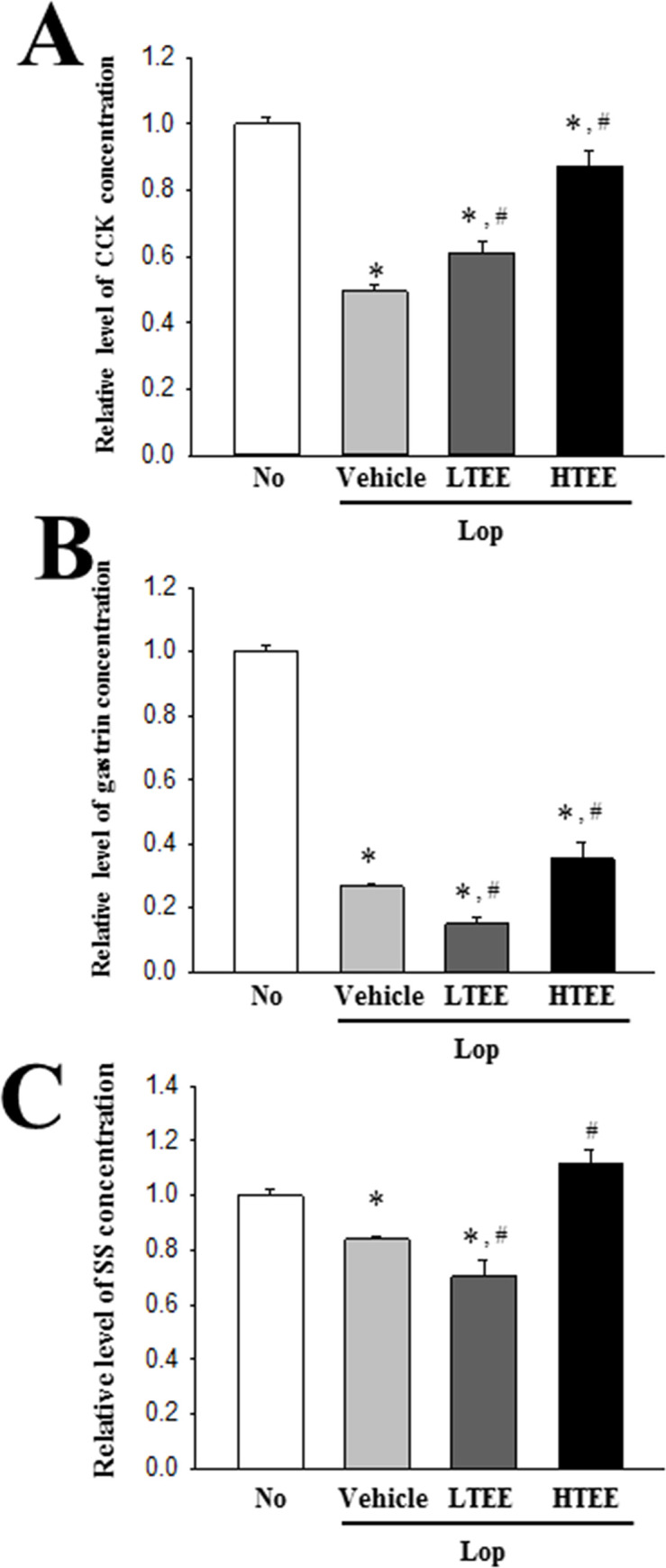
Concentrations of GI hormones in Lop+TEE treated constipation rats. The concentration of (A) CCK, (B) gastrin, and (C) SS was measured in the transverse colon homogenate by an enzyme-linked immunosorbent assay. The minimum detectable concentration of each kit is 0.1–1,000 pg/mL of CCK, 0.312–20 pg/mL of gastrin, and 4.7–300 pg/mL of SS. Five to six rats per group were used in preparation of tissue homogenate, and hormone level was assayed in duplicate in each sample. The data are reported as the mean ± SD. *, p<0.05 compared to the No treated group. ^#^, p<0.05 compared to the Lop+Vehicle treated group. Abbreviations: Lop, Loperamide; GI, Gastrointestinal; CCK, Cholecystokinin; SS, somatostatin.

To investigate whether the defecation stimulation effects of TEE are accompanied by alterations in regulating the secretion of GI hormones of constipation rats, we measured the concentrations of CCK, gastrin and SS in the colon homogenate of Lop-induced constipation rats after TEE treatment. Exposure to Lop resulted in decreased concentration of the three GI hormones, as compared to the No treated group. However, these concentrations were significantly increased in the Lop+TEE treated groups, although the increase rate was varied (Fig 8). These data indicate that TEE administration stimulates the secretion of GI hormones in Lop-induced constipation rats.

### Effect of TEE administration on the downstream signaling pathway of mAChRs

**Fig 9:**
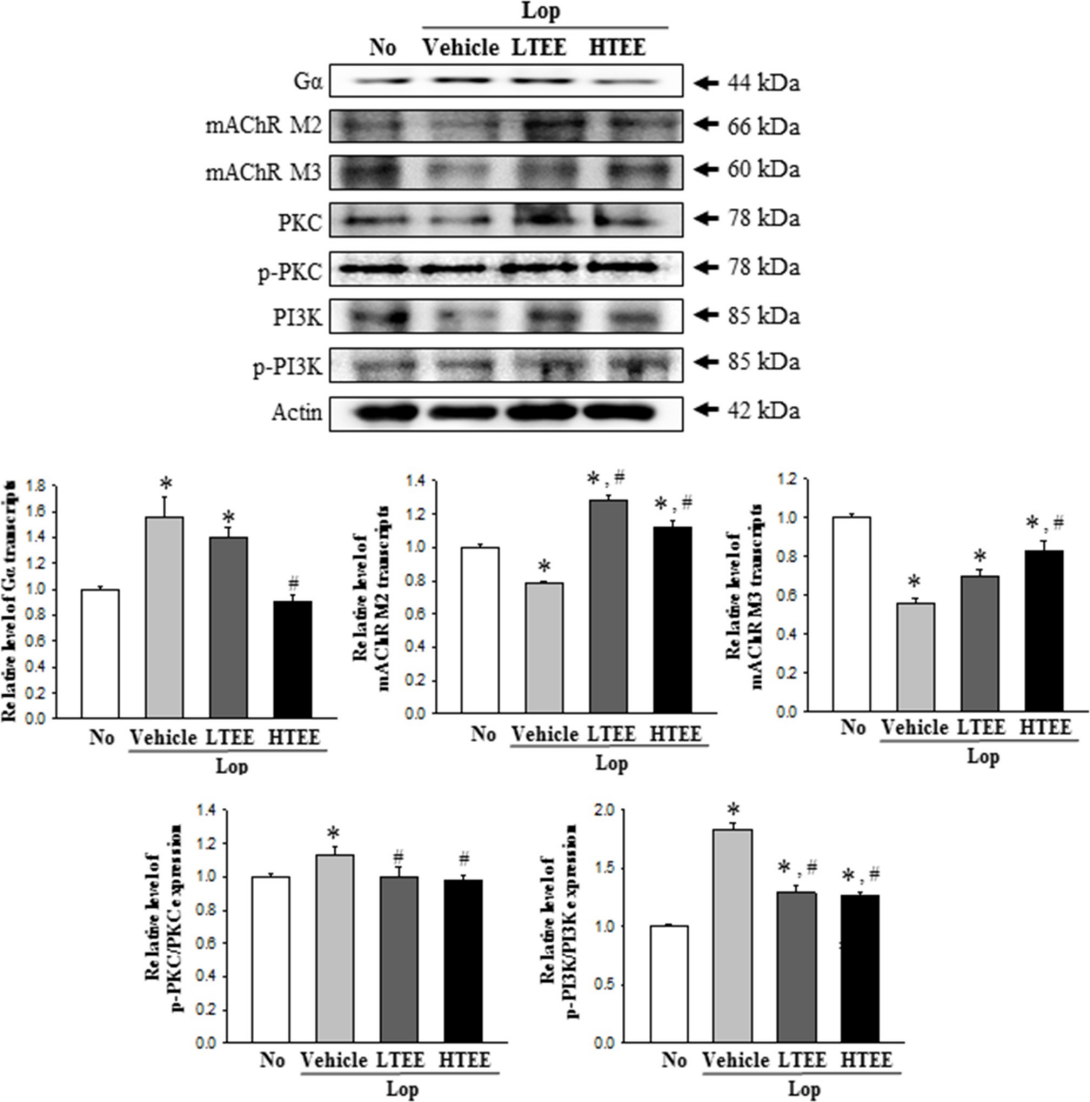
Expressions of mAChRs and key mediators within their downstream signaling pathway in Lop+TEE treated constipation rats. Expression levels of mAChRs and key mediators, including mAChR M2, mAChR M3, Gα, PKC, p-PKC, PI3K and p-PI3K, in the mAChR M2 and M3 signaling pathway were measured by Western blot analysis using specific primary antibodies and HRP-labeled anti-rabbit IgG antibody. After the intensity of each band was determined using an imaging densitometer, relative levels of the seven proteins were calculated based on the intensity of actin. Four to six rats per group were used in the preparation of the total tissue homogenate, and Western blot analyses were assayed in duplicate in each sample. The data are reported as the mean ± SD. *, p<0.05 compared to the No treated group. ^#^, p<0.05 compared to the Lop+Vehicle treated group. Abbreviations: Lop, Loperamide; mAChR, muscarinic acetylcholine receptors; PKC, Protein kinase C; PI3K, Phosphoinositide 3-kinases; HRP, Horseradish peroxidase; IgG, Immunoglobulin G.

Finally, to investigate whether the defecation stimulation effects of TEE are associated with the regulation of the mAChR signaling pathway in transverse colon of constipation rats, alterations in the expressions of mAChR M2, mAChR M3, Gα, PKC, p-PKC, PI3K and p-PI3K were measured in the transverse colons of subset groups. Decreased levels of mAChR M2 and mAChR M3 in the Lop+Vehicle treated group were remarkably increased after TEE administration. Similar alterations were observed in the expression of key mediators of the mAChR downstream signaling pathway. The phosphorylation levels of PKC and PI3K were dramatically, but not completely, recovered in the Lop+TEE treated groups, relative to the Lop+Vehicle treated group. However, a reverse pattern was observed for alterations of the mAChR signaling pathway with respect to Gα expression. Increased levels of Gα expression were recovered in the Lop+TEE treated groups, as compared to No treated group (Fig 9). Results of this study suggest that the defecation stimulation effects of TEE are accompanied with recovery of the down-regulation of mAChRs expression and their downstream signals in transverse colons of constipated SD rats.

## Discussion

Antioxidants help in reducing various chronic diseases, including cardiovascular disease, brain dysfunction, cancer, immune system decline, aging, and cataracts, due to their crucial role in the prevention of free radical formation [20]. Some natural products with high antioxidant activity have attracted great attention as novel strategies to overcome the disadvantages of synthetic antioxidants. Of these, only a few products have exerted both laxative effect and antioxidant properties in constipation models [5]. Since several tannin-containing natural products have been implicated in alleviating the symptoms of constipation [8, 10–12], we undertook to investigate the antioxidative activity and laxative effects of TEE in Lop-induced constipated SD rats. Results of the current study provide the first evidence that TEE induces laxative effects, including elevation of stool excretion and recovery of histological changes induced by Lop injection in the transverse colon. We believe our data to be the first to demonstrate that laxative effects of TEE may be associated with antioxidant effects, such as upregulation of antioxidant enzymes, downregulation of transcription factors, and suppression of ROS production.

*E*. *cava* used in this study is widely distributed as a perennial brown alga along the coastal area of Korea and Japan as well as traditionally used as foods and functional foods [21]. It has a variety of bioactive compounds and derivatives including the key compound phlorotannins, as well as sulphated polysaccharides, peptides, carotenoids, and fucoidans [22, 23]. Based on the above composition, therapeutic effects of *E*. *cava* extract have been examined for their antioxidant properties [24, 25], anti-human immunodeficiency virus type-1 (HIV-1) activity [26], apoptosis induction activity [27], Angiotensin 1-converting enzyme inhibitory effects [28], anti-inflammatory effects [29] and antidiabetic effects [30]. However, none of the *E*. *cava* extracts have been applied for the treatment of chronic constipation. In the current study, TEE treatment induced the recovery of most constipation symptoms, including stool parameters, gastrointestinal transit, histopathological structure of the transverse colon, and mAChR signaling pathway. These results provide the first evidence for the novel function of TEE, and indicates the potential of *E*. *cava* as a beneficial health application in functional foods and pharmaceutical industries. However, further research using different extracts of *E*. *cava* is required to determine the main compound active in efficacy on the antioxidant activity and laxative effects. Especially, eckol, a precursor compound of phlorotannins contained in TEE, can be considered an important candidate due to its numerous known biological activities including antioxidant [31], anti-inflammatory [32], hepatoprotective [33], neuroprotective [34], anti-obesity [35], and anti-hypertensive [36]. Moreover, eckol is majorly produced in brown algae (including Ecklonia species) and red algae [37].

The current study determined the antioxidant activity of TEE in pRISMCs and transverse colon of Lop-induced constipation rats. TEE, an aqueous extract of *E*. *cava*, significantly enhanced SOD and CAT activity, as well as decreased the ROS production. A similar effect was observed in previous studies that evaluated antioxidant effects of various extracts or compounds derived from *E*. *cava*, although the type of extracts analyzed and the assay factors were varied. Celluclast hydrolysate of *E*. *cava* effectively scavenged DPPH free radicals (73%), while 70% methanol extract of *E*. *cava* exhibited high activity in all antioxidant assays including DPPH, superoxide anion, hydrogen peroxide, hydroxyl radical, nitric oxide, ferrous ion chelating, reducing power and lipid peroxidation inhibition [38, 39]. Also, high radical scavenging activity against DPPH and alkyl, and inhibition of DNA damage induced by H_2_O_2_ were detected in pyrogallol-phloroglucinol-6,6’-bieckol (PPB) isolated from *E*. *cava* [40]. The fucoidan extract from *E*. *cava* (FEC) protected H_2_O_2_-induced cellular damage in neuronal cells, through the regulation of ABTS radical scavenging activity, MDA-inhibitory effect, and intracellular ROS content [41]. Therefore, the results of the present study show additional evidence that an aqueous extract of *E*. *cava* successfully induces antioxidant effects in intestinal cells and tissues.

Significant laxative effects, based on the ability to increase the number and weight of stools in the constipation model, were induced by various natural products including *Aloe ferox* Mill. [42], *Ficus carica* paste [43], and *L*. *platyphylla* [9]. Of these, several plant extracts such as *M*. *micrantha* Mull. Arg. [10]; *S*. *macranthera* [11], *Galla rhois* (GEGR) [8] and *U*. *indica* Kunth. [12] contain tannin, which is reported to possess strong antioxidant activity through chelation of metal ions and interfering with the reaction step of the Fenton reaction [44]. These extracts dramatically relieved the symptoms of constipation, including stool-related factors, bowel movement, histopathological structure, mucin secretion and mAChR signaling pathway, in the Lop-induced constipation model [10, 42]. Especially, the aqueous extract of galla rhois containing high level of tannin (69%), exhibited laxative effects in the Lop-induced constipation model [8]. However, none of the previous studies examined the association of the antioxidant capacity of tannin-contained extracts and the laxative effect. Hence, in the current study, we examined the antioxidant activity and laxative effects of another tannin-enriched extract to verify the correlation between oxidative stress and constipation. TEE contains 33% tannin, which is relatively high, although lesser than galla rhois. Furthermore, our results indicate the possibility that TEE containing high concentrations of tannin is a novel natural product with high laxative effects and antioxidant activity, although further studies are required to understand how tannin regulates the suppression of oxidative stress and motility of the gastrointestinal tract.

Meanwhile, the effects of natural products on the improvement of constipation were studied at various concentrations. Many natural products including *A*. *ferox* Mill., *Lepidium sativum*, *Fumaria parviflora*, *Phyllanthus emblica* and *Viola betonicifolia* were effective at concentration below 300 mg/kg in mice and rats model with constipation [42, 45–48]. But, few natural products such as *L*. *platyphylla* and red *L*. *platyphylla* were single treated into Lop-treated SD rats at 1,000 mg/kg to verify the laxative effects [9, 49]. Furthermore, other groups were treated with wide range concentrations. Herbal mixture of *L*. *platyphylla*, *Glycyrrhiza uralensis*, and *Cinnamomum cassia* were effective at 100, 300 and 600 mg/kg in lop-induced constipation of SD rats [50], while three doses of galla rhois (250, 500 and 1,000 mg/kg) were orally administered to the same model at one time [8]. In this study, 200 and 400 mg/kg of TEE were used as the optimal concentration, based on the concentration of natural products with laxative activity used in a previous study. This dose was the equivalent to 32.4 and 64.8 mg/kg daily dose in humans, according to the Food and Drug Administration in USA (Guidance for Industry Estimating the Maximum Safe Starting Dose in Initial Clinical Trials for Therapeutics in Adult Healthy Volunteers). However, the present study provides limited information for dose-dependent effects since only two dose of TEE were treated into Lop-induced model. Therefore, multidose analyses and molecular mechanism studies will be need to clarify the laxative role of TEE.

Taken together, our study results indicate that TEE induces the recovery of stool parameters, gastrointestinal mobility, histopathological alterations of the transverse colon, mucin secretion, GI hormone concentration and mAChR signaling pathway in Lop-induced constipation rats. Especially, these results provide evidence that the laxative effects of TEE are tightly correlated with the upregulation of antioxidant enzymes, downregulation of transcription factor and suppression of ROS production. Furthermore, these findings indicate that tannin-containing herbal plants are potential therapeutic candidates for the treatment of constipation, although some additional studies are required to verify the molecular mechanisms involved.

## Supporting information

S1 Data (PDF)
