# Supplementary material for: Antioxidant activity and laxative effects of tannin-enriched extract of Ecklonia cava in loperamide-induced constipation of SD rats
Source: PLoS One. 2021 Feb 24;16(2):e0246363. doi: 10.1371/journal.pone.0246363 (PMC7904174; doi:10.1371/journal.pone.0246363)
Supplement: S1 Data — (PDF) [file pone.0246363.s001.pdf]

Supporting Materials 1
Western blot image for SOD proteins in Fig. 2

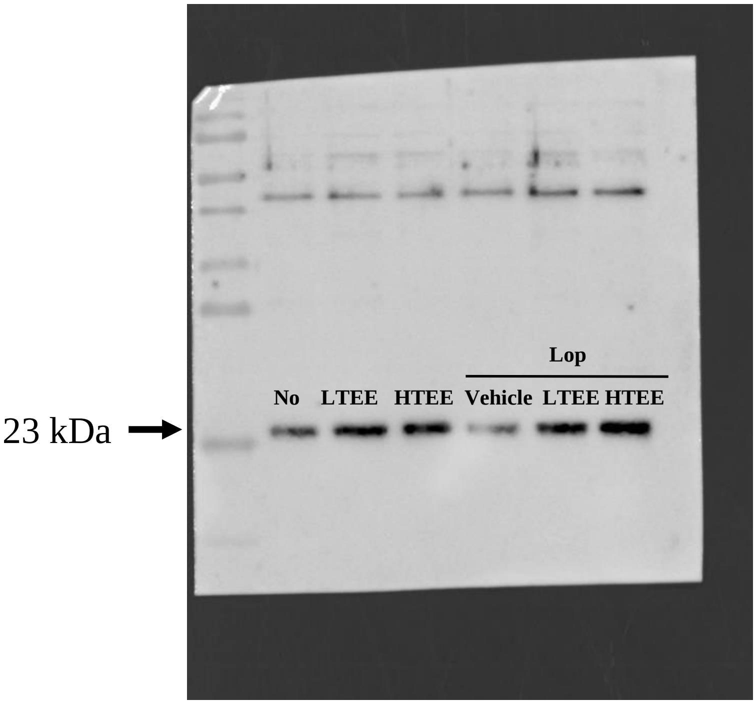

Expression of SOD in Lop+TEE treated pRISMC. Briefly, total lysates of pRISMC were prepared using homogenizer and separated in SDS-PAGE gel. The expression level of actin protein with colon homogenate transferred on the membrane was determined by HRP-conjugated anti-rabbit IgG antibody during Western blot analysis.

Supporting Materials 2
Western blot image for Nrf2 proteins in Fig. 2

110 kDa →

Expression of Nrf2 in Lop+TEE treated pRISMC. Briefly, total lysates of pRISMC were prepared using homogenizer and separated in SDS-PAGE gel. The expression level of actin protein with colon homogenate transferred on the membrane was determined by HRP-conjugated anti-rabbit IgG antibody during Western blot analysis.

Supporting Materials 3
Western blot image for p-Nrf2 proteins in Fig. 2

Lop

No LTEE HTEE Vehicle LTEE HTEE

110 kDa →

Expression of p-Nrf2 in Lop+TEE treated pRISMC. Briefly, total lysates of pRISMC were prepared using homogenizer and separated in SDS-PAGE gel. The expression level of actin protein with colon homogenate transferred on the membrane was determined by HRP-conjugated anti-rabbit IgG antibody during Western blot analysis.

Supporting Materials 4
Western blot image for Actin proteins in Fig. 2

42 kDa →

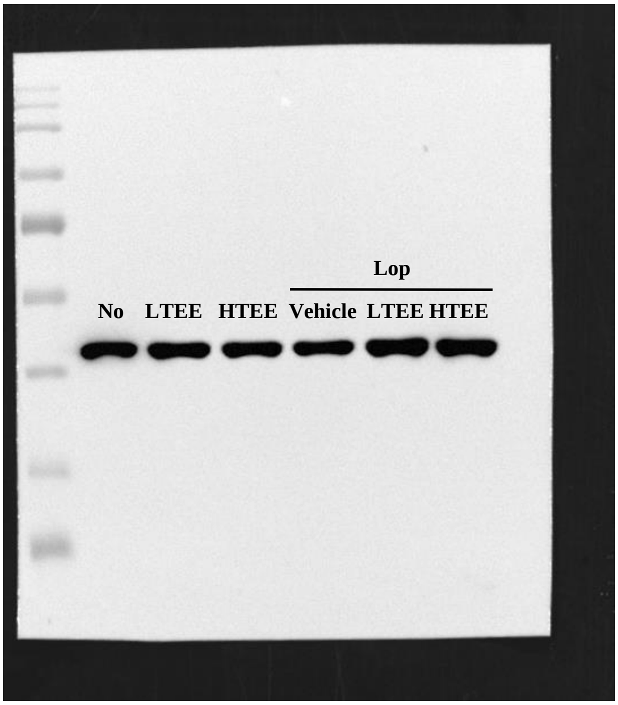

Expression of Actin in Lop+TEE treated pRISMC. Briefly, total lysates of pRISMC were prepared using homogenizer and separated in SDS-PAGE gel. The expression level of actin protein with colon homogenate transferred on the membrane was determined by HRP-conjugated anti-rabbit IgG antibody during Western blot analysis.

Supporting Materials 5
Western blot image for SOD proteins in Fig. 3

23 kDa

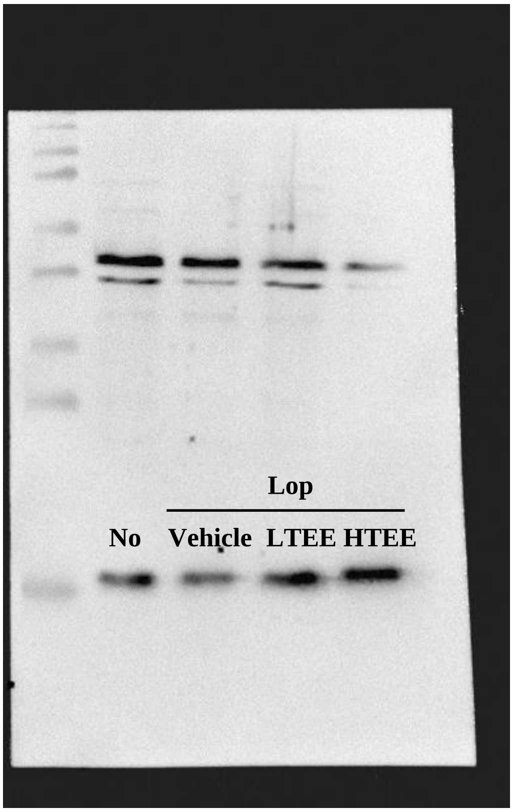

Expression of SOD in Lop+TEE treated rat colon. Briefly, total lysates of colon were prepared using homogenizer and separated in SDS-PAGE gel. The expression level of actin protein with colon homogenate transferred on the membrane was determined by HRP-conjugated anti-rabbit IgG antibody during Western blot analysis.

Supporting Materials 6
Western blot image for Actin proteins in Fig. 3

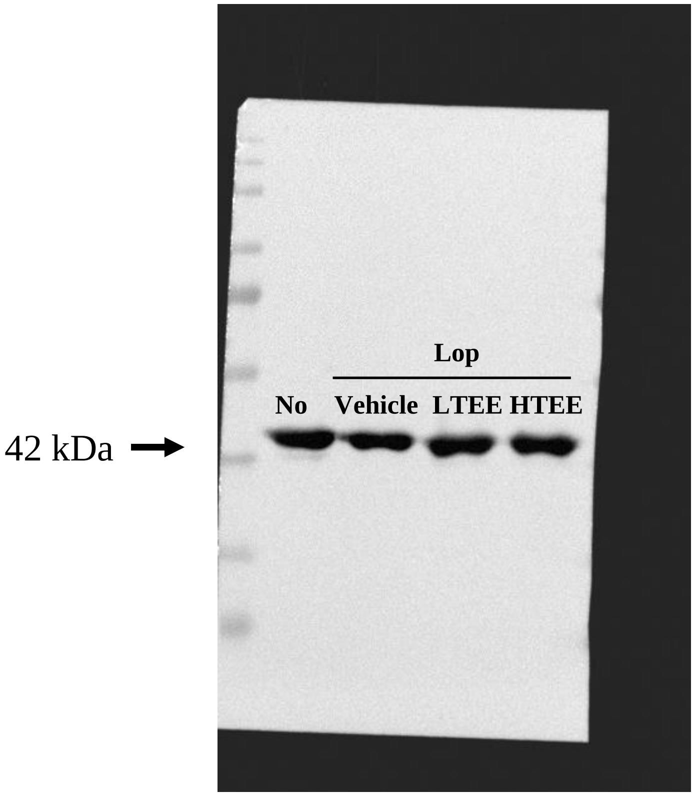

Expression of Actin in Lop+TEE treated rat colon. Briefly, total lysates of colon were prepared using homogenizer and separated in SDS-PAGE gel. The expression level of actin protein with colon homogenate transferred on the membrane was determined by HRP-conjugated anti-rabbit IgG antibody during Western blot analysis.

Supporting Materials 7
Western blot image for Nrf2 proteins in Fig. 3

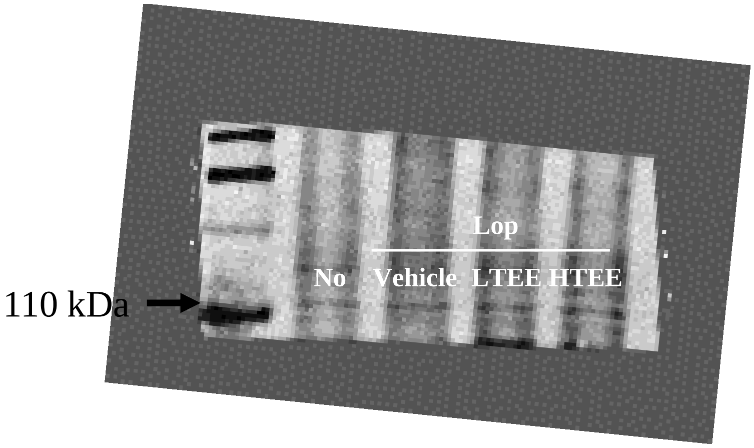

Expression of Nrf2 in Lop+TEE treated rat colon. Briefly, total lysates of colon were prepared using homogenizer and separated in SDS-PAGE gel. The expression level of actin protein with colon homogenate transferred on the membrane was determined by HRP-conjugated anti-rabbit IgG antibody during Western blot analysis.

Supporting Materials 8
Western blot image for p-Nrf2 proteins in Fig. 3

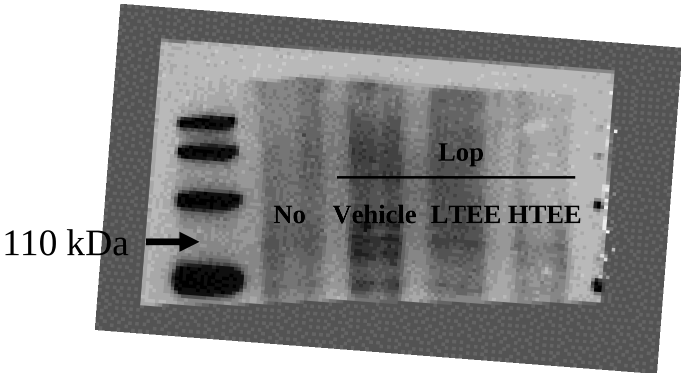

Expression of p-Nrf2 in Lop+TEE treated rat colon. Briefly, total lysates of colon were prepared using homogenizer and separated in SDS-PAGE gel. The expression level of actin protein with colon homogenate transferred on the membrane was determined by HRP-conjugated anti-rabbit IgG antibody during Western blot analysis.

Supporting Materials 9
Western blot image for Actin proteins in Fig. 3

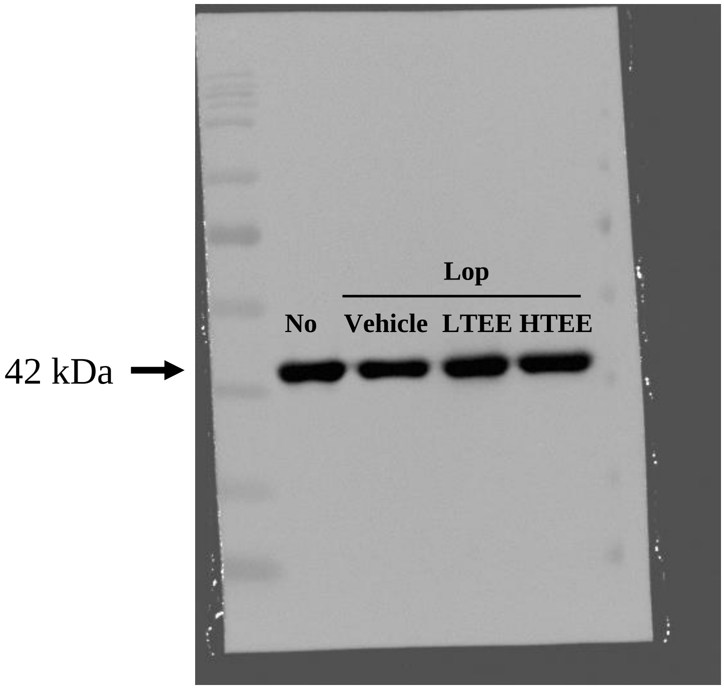

Expression of Actin in Lop+TEE treated rat colon. Briefly, total lysates of colon were prepared using homogenizer and separated in SDS-PAGE gel. The expression level of actin protein with colon homogenate transferred on the membrane was determined by HRP-conjugated anti-rabbit IgG antibody during Western blot analysis.

Supporting Materials 10
Western blot image for Gα proteins in Fig. 9

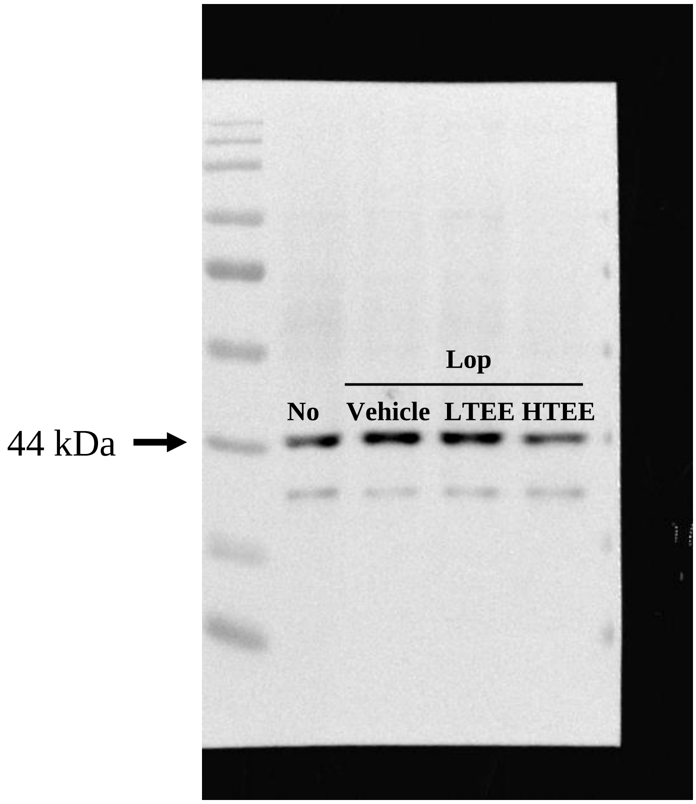

Expression of Gα in Lop+TEE treated rat colon. Briefly, total lysates of colon were prepared using homogenizer and separated in SDS-PAGE gel. The expression level of actin protein with colon homogenate transferred on the membrane was determined by HRP-conjugated anti-rabbit IgG antibody during Western blot analysis.

Supporting Materials 11
Western blot image for mAChR M2 proteins in Fig. 9

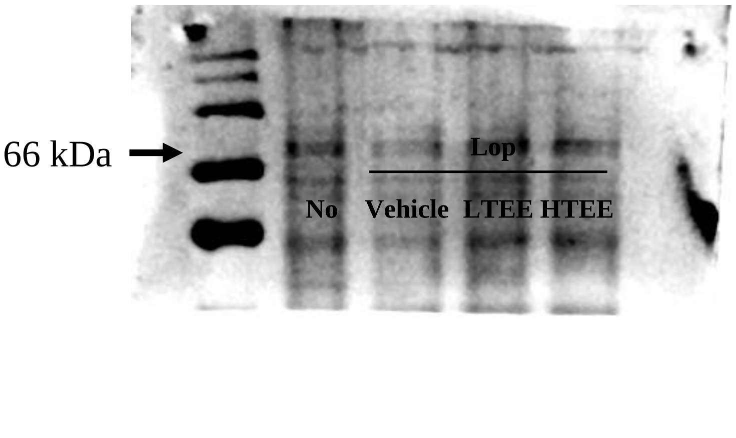

Expression of mAChR M2 in Lop+TEE treated rat colon. Briefly, total lysates of colon were prepared using homogenizer and separated in SDS-PAGE gel. The expression level of actin protein with colon homogenate transferred on the membrane was determined by HRP-conjugated anti-rabbit IgG antibody during Western blot analysis.

Supporting Materials 12
Western blot image for mAChR M3 proteins in Fig. 9

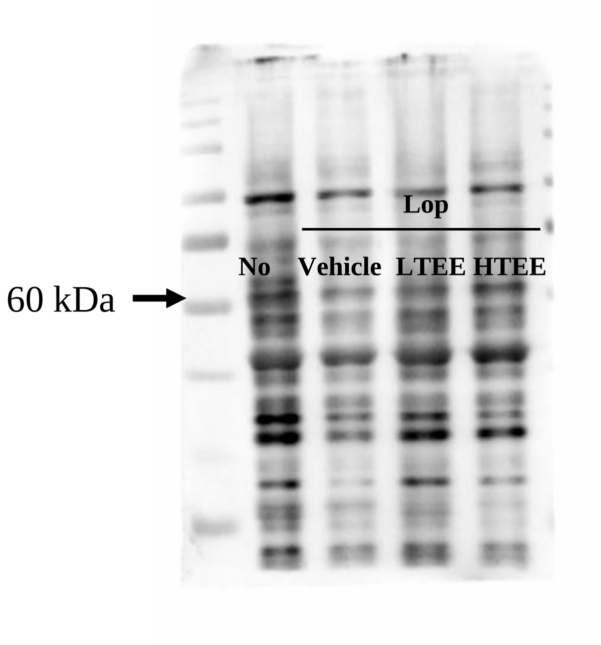

Expression of mAChR M3 in Lop+TEE treated rat colon. Briefly, total lysates of colon were prepared using homogenizer and separated in SDS-PAGE gel. The expression level of actin protein with colon homogenate transferred on the membrane was determined by HRP-conjugated anti-rabbit IgG antibody during Western blot analysis.

Supporting Materials 13
Western blot image for PKC proteins in Fig. 9

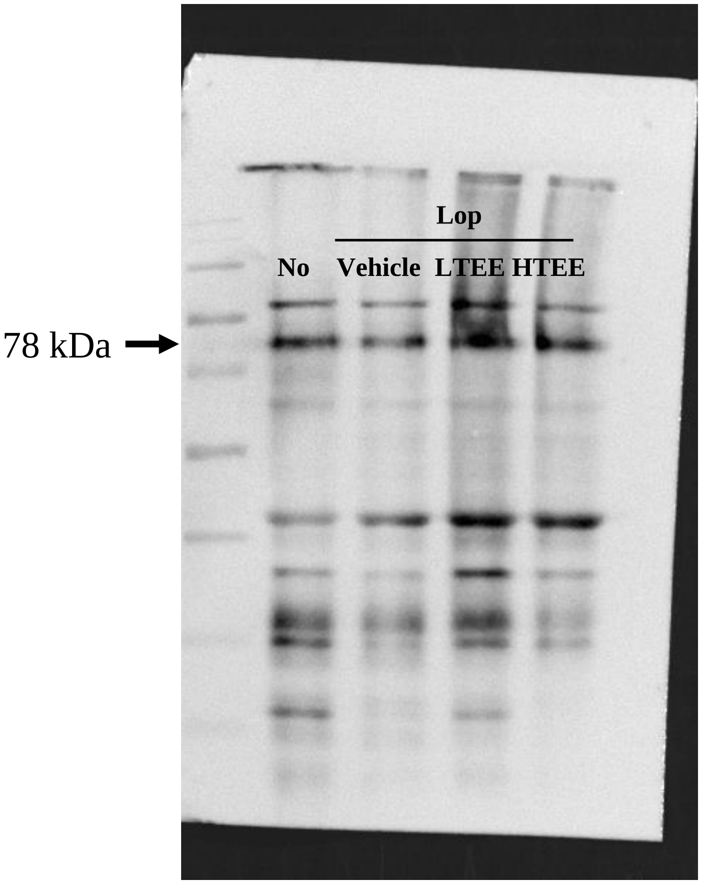

Expression of PKC in Lop+TEE treated rat colon. Briefly, total lysates of colon were prepared using homogenizer and separated in SDS-PAGE gel. The expression level of actin protein with colon homogenate transferred on the membrane was determined by HRP-conjugated anti-rabbit IgG antibody during Western blot analysis.

Supporting Materials 14
Western blot image for p-PKC proteins in Fig. 9

78 kDa →

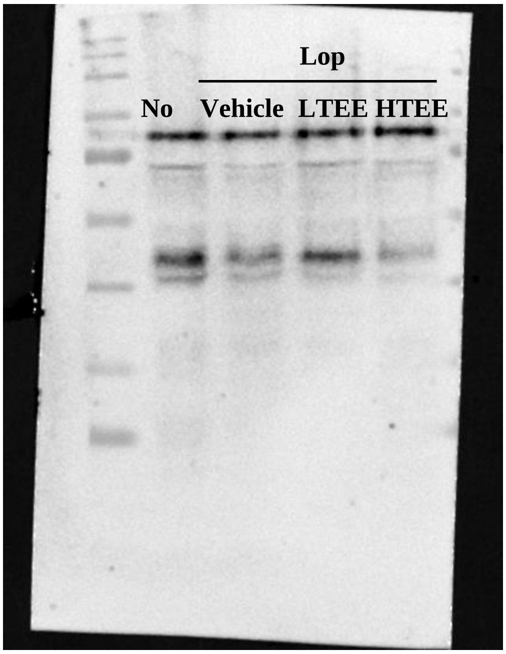

Expression of p-PKC in Lop+TEE treated rat colon. Briefly, total lysates of colon were prepared using homogenizer and separated in SDS-PAGE gel. The expression level of actin protein with colon homogenate transferred on the membrane was determined by HRP-conjugated anti-rabbit IgG antibody during Western blot analysis.

Supporting Materials 15
Western blot image for PI3K proteins in Fig. 9

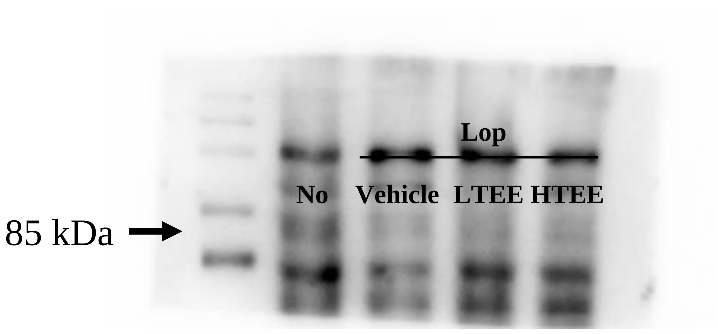

Expression of PI3K in Lop+TEE treated rat colon. Briefly, total lysates of colon were prepared using homogenizer and separated in SDS-PAGE gel. The expression level of actin protein with colon homogenate transferred on the membrane was determined by HRP-conjugated anti-rabbit IgG antibody during Western blot analysis.

Supporting Materials 16
Western blot image for p-PI3K proteins in Fig. 9

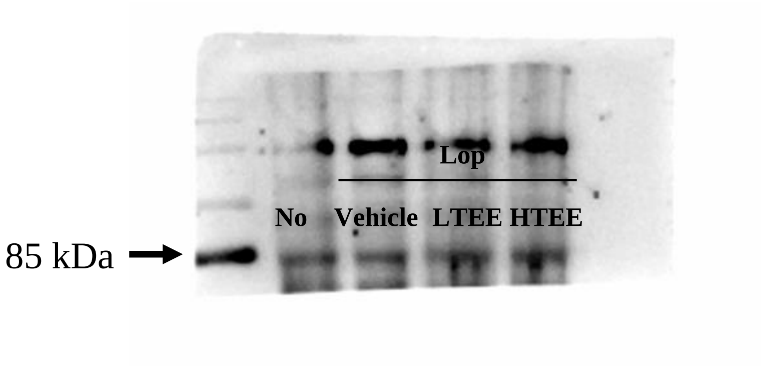

Expression of p-PI3K in Lop+TEE treated rat colon. Briefly, total lysates of colon were prepared using homogenizer and separated in SDS-PAGE gel. The expression level of actin protein with colon homogenate transferred on the membrane was determined by HRP-conjugated anti-rabbit IgG antibody during Western blot analysis.

Supporting Materials 17
Western blot image for Actin proteins in Fig. 9

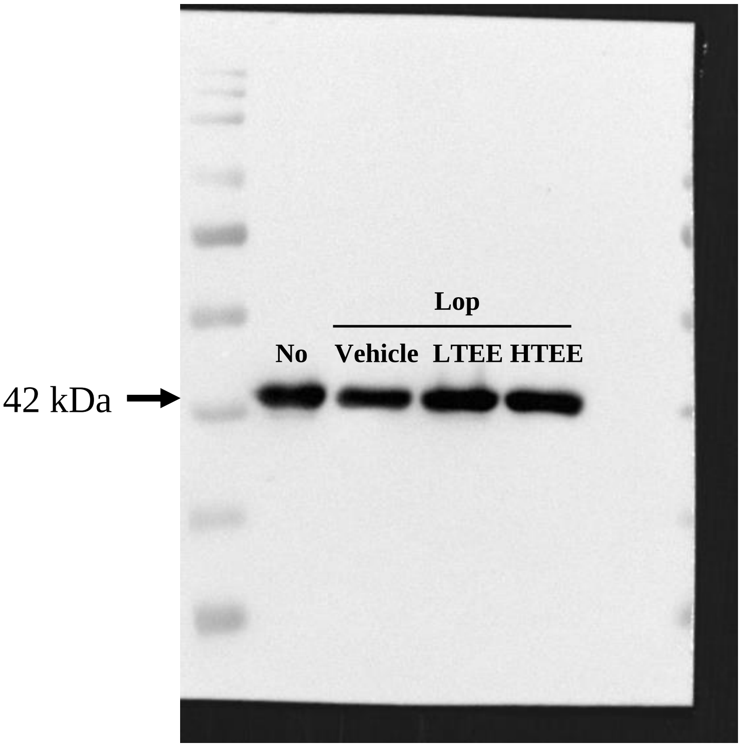

Expression of Actin in Lop+TEE treated rat colon. Briefly, total lysates of colon were prepared using homogenizer and separated in SDS-PAGE gel. The expression level of actin protein with colon homogenate transferred on the membrane was determined by HRP-conjugated anti-rabbit IgG antibody during Western blot analysis.
