# Supplementary material for: Antioxidant activity and laxative effects of tannin-enriched extract of Ecklonia cava in loperamide-induced constipation of SD rats
Source: PLoS One. 2021 Feb 24;16(2):e0246363. doi: 10.1371/journal.pone.0246363 (PMC7904174; doi:10.1371/journal.pone.0246363)
Supplement: Peer review history

# Peer review: Decision Letter 0

**DOI:** 10.1371/journal.pone.0246363.r001

**Authors:** Saikat Dewanjee

1 Sep 2020

PONE-D-20-24159

Anti-oxidant activity and laxative effects of tannin-enriched extract of Ecklonia cava in loperamide-induced constipation of SD rats

PLOS ONE

Dear Dr. Hwang,

Thank you for submitting your manuscript to PLOS ONE. After careful consideration, we feel that it has merit but does not fully meet PLOS ONE’s publication criteria as it currently stands. Therefore, we invite you to submit a revised version of the manuscript that addresses the points raised during the review process.

The manuscript needs extensive revision considering reviewer comments. In addition to these comments, authors must consider the following points

1. Authors used an extensively high dose range of 250-1000 mg/kg in the experiment, what is the justification. Is it scientifically sound to use 1000 mg/kg?

2. Authors are suggested to include the data of all three doses in the manuscript to depict the dose-dependent effect.

3. Blot images are not convincing and often misleading. These must be replaced by suitable blot images. In addition, authors are requested to submit uncropped and unedited full-length blot images as a supplementary document.

4. The quantified P-Nrf-2 expression is not convincing.

5. WELo WEHi??? Authors did not mention either in the body of the manuscript or in the figure caption.

Please submit your revised manuscript by Oct 16 2020 11:59PM. If you will need more time than this to complete your revisions, please reply to this message or contact the journal office at plosone@plos.org. When you're ready to submit your revision, log on to https://www.editorialmanager.com/pone/ and select the 'Submissions Needing Revision' folder to locate your manuscript file.

Please include the following items when submitting your revised manuscript:

- A rebuttal letter that responds to each point raised by the academic editor and reviewer(s). You should upload this letter as a separate file labeled 'Response to Reviewers'.
- A marked-up copy of your manuscript that highlights changes made to the original version. You should upload this as a separate file labeled 'Revised Manuscript with Track Changes'.
- An unmarked version of your revised paper without tracked changes. You should upload this as a separate file labeled 'Manuscript'.

If you would like to make changes to your financial disclosure, please include your updated statement in your cover letter. Guidelines for resubmitting your figure files are available below the reviewer comments at the end of this letter.

If applicable, we recommend that you deposit your laboratory protocols in protocols.io to enhance the reproducibility of your results. Protocols.io assigns your protocol its own identifier (DOI) so that it can be cited independently in the future. For instructions see: http://journals.plos.org/plosone/s/submission-guidelines#loc-laboratory-protocols

We look forward to receiving your revised manuscript.

Kind regards,

Saikat Dewanjee

Academic Editor

PLOS ONE

Journal Requirements:

When submitting your revision, we need you to address these additional requirements.

1. Please ensure that your manuscript meets PLOS ONE's style requirements, including those for file naming. The PLOS ONE style templates can be found at

https://journals.plos.org/plosone/s/file?id=wjVg/PLOSOne_formatting_sample_main_body.pdf and

https://journals.plos.org/plosone/s/file?id=ba62/PLOSOne_formatting_sample_title_authors_affiliations.pdf

2. As part of your revisions we kindly request that you update your Methods section with additional details pertaining to the extract that you investigated (Ecklonia cava) and your animal research procedures. Please address the following: (1) Name and Location/address of vendor, lot number, name of product (if it  differs from name of herbal extract),  sensory characters such as taste, appearance, odor, texture and so forth. (2) Report of all components or ingredients of the product (this can be listed in a supplemental document if you prefer; (3) Details of quality assurance/characterization; (4) Description of experimental design: including justification for in vivo model used (why rats and not mice?); (5) All methods undertaken to minimize potential pain and distress; (6) description of adverse events experienced by the animals (sickness? death? etc.). (7) If any of the rats died prior to experimental endpoint, state cause of death.

3. Thank you for stating the following in the Acknowledgments Section of your manuscript:

"This research was supported by Basic Science Research Program through the National Research Foundation of Korea (NRF) funded by the Ministry of Education (2020R1l1A1A01052277). This

study was financially supported by the『2020 Post-Doc. Development Program』of Pusan National

University."

We note that you have provided funding information that is not currently declared in your Funding Statement. However, funding information should not appear in the Acknowledgments section or other areas of your manuscript. We will only publish funding information present in the Funding Statement section of the online submission form.

Please remove any funding-related text from the manuscript and let us know how you would like to update your Funding Statement. Currently, your Funding Statement reads as follows:

"The funders had no role in study design, data collection and analysis, decision to publish, or preparation of the manuscript."

4. PLOS ONE now requires that authors provide the original uncropped and unadjusted images underlying all blot or gel results reported in a submission’s figures or Supporting Information files. This policy and the journal’s other requirements for blot/gel reporting and figure preparation are described in detail at https://journals.plos.org/plosone/s/figures#loc-blot-and-gel-reporting-requirements and https://journals.plos.org/plosone/s/figures#loc-preparing-figures-from-image-files. When you submit your revised manuscript, please ensure that your figures adhere fully to these guidelines and provide the original underlying images for all blot or gel data reported in your submission. See the following link for instructions on providing the original image data: https://journals.plos.org/plosone/s/figures#loc-original-images-for-blots-and-gels.

In your cover letter, please note whether your blot/gel image data are in Supporting Information or posted at a public data repository, provide the repository URL if relevant, and provide specific details as to which raw blot/gel images, if any, are not available. Email us at plosone@plos.org if you have any questions.

5. Please upload a new copy of Figure xxxx as the detail is not clear. Please follow the link for more information: https://blogs.plos.org/plos/2019/06/looking-good-tips-for-creating-your-plos-figures-graphics/

[Note: HTML markup is below. Please do not edit.]

Reviewers' comments:

Reviewer's Responses to Questions

**Comments to the Author**

1. Is the manuscript technically sound, and do the data support the conclusions?

The manuscript must describe a technically sound piece of scientific research with data that supports the conclusions. Experiments must have been conducted rigorously, with appropriate controls, replication, and sample sizes. The conclusions must be drawn appropriately based on the data presented. 

Reviewer #1: Yes

Reviewer #2: Partly

**********

2. Has the statistical analysis been performed appropriately and rigorously? 

Reviewer #1: Yes

Reviewer #2: Yes

**********

3. Have the authors made all data underlying the findings in their manuscript fully available?

The PLOS Data policy requires authors to make all data underlying the findings described in their manuscript fully available without restriction, with rare exception (please refer to the Data Availability Statement in the manuscript PDF file). The data should be provided as part of the manuscript or its supporting information, or deposited to a public repository. For example, in addition to summary statistics, the data points behind means, medians and variance measures should be available. If there are restrictions on publicly sharing data—e.g. participant privacy or use of data from a third party—those must be specified.

Reviewer #1: Yes

Reviewer #2: Yes

**********

4. Is the manuscript presented in an intelligible fashion and written in standard English?

PLOS ONE does not copyedit accepted manuscripts, so the language in submitted articles must be clear, correct, and unambiguous. Any typographical or grammatical errors should be corrected at revision, so please note any specific errors here.

Reviewer #1: Yes

Reviewer #2: Yes

**********

5. Review Comments to the Author

Please use the space provided to explain your answers to the questions above. You may also include additional comments for the author, including concerns about dual publication, research ethics, or publication ethics. (Please upload your review as an attachment if it exceeds 20,000 characters)

Reviewer #1: The authors examined alterations after exposure to TEE, which is a tannin-enriched extracts of Ecklonia cava on constipation phenotypes, muscarinic cholinergic regulation, and oxidative stress responses in the transverse colons of SD rats with loperamide (Lop)-induced constipation. They found TEE treatment increases, improvements in ROS production, SOD expression and Nrf2 phosphorylation in primary smooth muscles of rat intestine cells (pRISMCs) and transverse colon of constipation model. And finally the showed TEE induces the recovery of stool parameters, gastrointestinal mobility, histopathological alterations of the transverse colon, mucin secretion, GI hormone concentration and mAChR signaling pathway in Lop-induced constipation rats. Overall the author’s methods, results, and discussion of the results and their potential implications seem appropriate for the data presented. With revisions to address the issues noted below, the study may be suitable for publication.

The authors should include the gender, and weight of SD rats employed in the study.

The group designing and experimental details are confusing. They should make it simpler with the inclusion of a flow chart indicating all details and timings.

They should clearly mention at what time point they sacrificed the animals to collect the colon samples.

The authors should provide the catalog number, and dilution details for the primary antibodies used in the WB experiment.

The authors should state whether the investigators were blinded/or randomization was performed while performing histopathological experiments.

They should also maintain the same font and font size throughout the manuscript.

Reviewer #2: The work done by JiEun Kimet al., and reported in the manuscript, entitled “Anti-oxidant activity and laxative effects of tannin-enriched 4 extract of Ecklonia cava in loperamide-induced constipation 5 of SD rats” has a proper scientific approach. There are few comments that need to be properly addressed before further progress of the manuscript.

1. In the manuscript author subdivided the animals into No group, Lop+Vehicle treated group, Lop+LTEE treated group, and Lop+HTEE treated group ( page no.8 line no. 180- page no.9 line no. 188). But in all of the figures they mentions groups as No, vehicle, WELo, WEHi. Can authors clarify the issue? The authors should do the necessary changes in the manuscript.

2. The authors should flowcytometrically measure the levels of ROS production in the manuscript.

3. The author should provide appropriate histological markings in Figure 6 A and Figure 7 A.

4. The authors should incorporate a graphical presentation of their findings to enrich the manuscript.

5. Author should go through the manuscript thoroughly to check the typographical errors and grammatical mistakes.

**********

6. PLOS authors have the option to publish the peer review history of their article (what does this mean?). If published, this will include your full peer review and any attached files.

If you choose “no”, your identity will remain anonymous but your review may still be made public.

**Do you want your identity to be public for this peer review?** For information about this choice, including consent withdrawal, please see our Privacy Policy.

Reviewer #1: **Yes: **Deep Sharma

Reviewer #2: No

[NOTE: If reviewer comments were submitted as an attachment file, they will be attached to this email and accessible via the submission site. Please log into your account, locate the manuscript record, and check for the action link "View Attachments". If this link does not appear, there are no attachment files.]

While revising your submission, please upload your figure files to the Preflight Analysis and Conversion Engine (PACE) digital diagnostic tool, https://pacev2.apexcovantage.com/. PACE helps ensure that figures meet PLOS requirements. To use PACE, you must first register as a user. Registration is free. Then, login and navigate to the UPLOAD tab, where you will find detailed instructions on how to use the tool. If you encounter any issues or have any questions when using PACE, please email PLOS at figures@plos.org. Please note that Supporting Information files do not need this step.

# Author comment: Author response to Decision Letter 0

**DOI:** 10.1371/journal.pone.0246363.r002

23 Dec 2020

Editor comments:

The manuscript needs extensive revision considering reviewer comments. In addition to these comments, authors must consider the following points.

1. Authors used an extensively high dose range of 250-1000 mg/kg in the experiment, what is the justification. Is it scientifically sound to use 1000 mg/kg?

☞ In this study, two dose of TEE (200 and 400 mg/kg), not 250-1,000 mg/kg, were treated into Lop-induced constipation models. Therefore, sentences related with above issue in materials and methods (line 171-173) have been deleted from our text to prevent confusion among readers.

“Laxative effects of TEE were measured as described in previous studies [8,12]. Briefly, 8-week-old SD rats (male, 270 g, n=28) were assigned to either a non-constipation group (No group, n=7) or a constipation group (n=21).”

 And, we further described the issue of concentration and limitations in Discussion section (line 487-500) as following; Please understand our situation.

“Meanwhile, the effects of natural products on the improvement of constipation were studied at various concentrations. Many natural products including Aloe ferox Mill., Lepidium sativum, Fumaria parviflora, Phyllanthus emblica and Viola betonicifolia were effective at concentration below 300 mg/kg in mice and rats model with constipation [42,45-48]. But, few natural products such as Liriope platyphylla and red Liriope platyphylla were single treated into Lop-treated SD rats at 1,000 mg/kg to verify the laxative effects [9,49]. Furthermore, other groups were treated with wide range concentrations. Herbal mixture of Liriope platyphylla, Glycyrrhiza uralensis, and Cinnamomum cassia were effective at 100, 300 and 600 mg/kg in lop-induced constipation of SD rats [50], while three doses of galla rhois (250, 500 and 1,000 mg/kg) were orally administered to the same model at one time [8]. In this study, 200 and 400 mg/kg of TEE were used as the optimal concentration, based on the concentration of natural products with laxative activity used in a previous study. This dose was the equivalent to 32.4 and 64.8 mg/kg daily dose in humans, according to the Food and Drug Administration in USA (Guidance for Industry Estimating the Maximum Safe Starting Dose in Initial Clinical Trials for Therapeutics in Adult Healthy Volunteers).”

2. Authors are suggested to include the data of all three doses in the manuscript to depict the dose-dependent effect.

☞ This is good point. At least three concentrations-treated groups need to be tested in order to prove a dose-dependent effects. But, our study did not show dose-dependent effects of TEE because only two doses TEE treated groups were analyzed. This is a limitation of our study. Therefore, that has been furthers described in Discussion (line 500-503) as following;

“However, the present study provides limited information for dose-dependent effects since only two dose of TEE were treated into Lop-induced model. Therefore, multidose analyses and molecular mechanism studies will be need to clarify the laxative role of TEE.”

3. Blot images are not convincing and often misleading. These must be replaced by suitable blot images. In addition, authors are requested to submit uncropped and unedited full-length blot images as a supplementary document.

☞ According to your comments, all western blot images have been replaced by suitable one. Also, the uncropped and unedited full-length blot images have been attached as a Supplementary data.

4. The quantified P-Nrf-2 expression is not convincing.

☞ This data has been replaced by new one. 

5. WELo WEHi??? Authors did not mention either in the body of the manuscript or in the figure caption.

☞ This is our mistake. All figure captions have been corrected based on manuscript. 

Reviewer's comments: 

Reviewer #1

The authors examined alterations after exposure to TEE, which is a tannin-enriched extracts of Ecklonia cava on constipation phenotypes, muscarinic cholinergic regulation, and oxidative stress responses in the transverse colons of SD rats with loperamide (Lop)-induced constipation. They found TEE treatment increases, improvements in ROS production, SOD expression and Nrf2 phosphorylation in primary smooth muscles of rat intestine cells (pRISMCs) and transverse colon of constipation model. And finally the showed TEE induces the recovery of stool parameters, gastrointestinal mobility, histopathological alterations of the transverse colon, mucin secretion, GI hormone concentration and mAChR signaling pathway in Lop-induced constipation rats. Overall the author’s methods, results, and discussion of the results and their potential implications seem appropriate for the data presented. With revisions to address the issues noted below, the study may be suitable for publication.1. The authors should include the gender, and weight of SD rats employed in the study.

☞ That has been included.

2. The group designing and experimental details are confusing. They should make it simpler with the inclusion of a flow chart indicating all details and timings.

☞ According to your comments, we have added experimental schedule as Figure 4A.

3. They should clearly mention at what time point they sacrificed the animals to collect the colon samples.

☞ According to your comments, we clearly corrected above issues in line 171-182 as following;

“Briefly, 8-week-old SD rats (male, 270 g, n=28) were assigned to either a non-constipation group (No group, n=7) or a constipation group (n=21). Subcutaneous injection of Lop (4 mg/kg weight) in 0.5% Tween 20 in saline was administered twice daily (9 a.m. and 6 p.m.) for 3 days to induce constipation, whereas the non-constipation group received 0.5% Tween 20 in saline alone under the same pattern. At 9 a.m. on the 4th day, the constipation group was divided into a Lop+Vehicle treated group (n=7), Lop+LTEE treated group (n=7), and Lop+HTEE treated group (n=7). The Lop+LTEE treated group was orally administered a single dose of 200 mg/kg body weight TEE, while the Lop+HTEE treated group received a single dose of 400 mg/kg body weight TEE. The Lop+Vehicle treated group was administered the same volume of 1× PBS under the same pattern. At 9 a.m. on the 5th day, total stools, urine, water and food were collected from the metabolic cage of each mice in subset group, and their levels were measured using appropriate methods. All SD rats were then euthanized using CO2 gas, after which transverse colon samples were acquired and stored at -70°C in Eppendorf tubes until assay (Fig 4A).”

4. The authors should provide the catalog number, and dilution details for the primary antibodies used in the WB experiment.

☞ That has been corrected.

5. The authors should state whether the investigators were blinded/or randomization was performed while performing histopathological experiments.

☞ That has been corrected in line 264-266 as following; 

“Furthermore, the histopathological analyses including slide preparation, H&E staining, mucin staining and microscopic observation were conducted randomly.”

6. They should also maintain the same font and font size throughout the manuscript.

☞ That has been corrected.

Reviewer #2

The work done by Ji Eun Kim et al., and reported in the manuscript, entitled “Anti-oxidant activity and laxative effects of tannin-enriched extract of Ecklonia cava in loperamide-induced constipation of SD rats” has a proper scientific approach. There are few comments that need to be properly addressed before further progress of the manuscript.1. In the manuscript author subdivided the animals into No group, Lop+Vehicle treated group, Lop+LTEE treated group, and Lop+HTEE treated group ( page no.8 line no. 180- page no.9 line no. 188). But in all of the figures they mentions groups as No, vehicle, WELo, WEHi. Can authors clarify the issue? The authors should do the necessary changes in the manuscript.

☞ This is our mistake. In the picture and body, the name of all groups have been corrected to constantly maintain as the same pattern.

2. The authors should flowcytometrically measure the levels of ROS production in the manuscript.

☞ This is good point. However, this analysis was not possible under the current conditions due to technical problems. To overcome this issue, the fluorescent level of DCFHDA stained cells were analyzed using a Fluorescence Microplate Reader in materials and methods (line 157-158) and the results (line 332-335) were added as Figure 2A.

“The DCFHDA fluorescence intensity was measured with a Fluorescence Microplate Reader (SpectraMax iD5, Molecular Devices) using 485 and 535 nm as excitation and emission filters respectively.”

“Antioxidant activity of TEE was similar to the inhibitory eﬀects against Lop-induced ROS production in pRISMCs, wherein a decreased number of DCF-DH stained cells and level of fluorescence intensity were obtained in the Lop+TEE treated groups (Fig 2A).”

3. The author should provide appropriate histological markings in Figure 6 A and Figure 7 A.

☞ That has been corrected.

4. The authors should incorporate a graphical presentation of their findings to enrich the manuscript. 

☞ According your comments, we have incorporated the graphical presentation for histopathological structure analysis in Figure 6 although most findings of our results have been incorporated as graphical presentation. 

5. Author should go through the manuscript thoroughly to check the typographical errors and grammatical mistakes.

☞ We carefully checked and reviewed all text based on your comments. The typographical errors and grammatical mistakes have been corrected in through all text.

# Peer review: Decision Letter 1

**DOI:** 10.1371/journal.pone.0246363.r003

**Authors:** Saikat Dewanjee

19 Jan 2021

Anti-oxidant activity and laxative effects of tannin-enriched extract of Ecklonia cava in loperamide-induced constipation of SD rats

PONE-D-20-24159R1

Dear Dr. Hwang,

We’re pleased to inform you that your manuscript has been judged scientifically suitable for publication and will be formally accepted for publication once it meets all outstanding technical requirements.

Within one week, you’ll receive an e-mail detailing the required amendments. When these have been addressed, you’ll receive a formal acceptance letter and your manuscript will be scheduled for publication.

An invoice for payment will follow shortly after the formal acceptance. To ensure an efficient process, please log into Editorial Manager at http://www.editorialmanager.com/pone/, click the 'Update My Information' link at the top of the page, and double check that your user information is up-to-date. If you have any billing related questions, please contact our Author Billing department directly at authorbilling@plos.org.

If your institution or institutions have a press office, please notify them about your upcoming paper to help maximize its impact. If they’ll be preparing press materials, please inform our press team as soon as possible -- no later than 48 hours after receiving the formal acceptance. Your manuscript will remain under strict press embargo until 2 pm Eastern Time on the date of publication. For more information, please contact onepress@plos.org.

Kind regards,

Saikat Dewanjee

Academic Editor

PLOS ONE

Additional Editor Comments (optional):

Reviewers' comments:

Reviewer's Responses to Questions

**Comments to the Author**

1. If the authors have adequately addressed your comments raised in a previous round of review and you feel that this manuscript is now acceptable for publication, you may indicate that here to bypass the “Comments to the Author” section, enter your conflict of interest statement in the “Confidential to Editor” section, and submit your "Accept" recommendation.

Reviewer #1: All comments have been addressed

Reviewer #2: All comments have been addressed

**********

2. Is the manuscript technically sound, and do the data support the conclusions?

The manuscript must describe a technically sound piece of scientific research with data that supports the conclusions. Experiments must have been conducted rigorously, with appropriate controls, replication, and sample sizes. The conclusions must be drawn appropriately based on the data presented. 

Reviewer #1: Yes

Reviewer #2: Yes

**********

3. Has the statistical analysis been performed appropriately and rigorously? 

Reviewer #1: Yes

Reviewer #2: Yes

**********

4. Have the authors made all data underlying the findings in their manuscript fully available?

The PLOS Data policy requires authors to make all data underlying the findings described in their manuscript fully available without restriction, with rare exception (please refer to the Data Availability Statement in the manuscript PDF file). The data should be provided as part of the manuscript or its supporting information, or deposited to a public repository. For example, in addition to summary statistics, the data points behind means, medians and variance measures should be available. If there are restrictions on publicly sharing data—e.g. participant privacy or use of data from a third party—those must be specified.

Reviewer #1: Yes

Reviewer #2: Yes

**********

5. Is the manuscript presented in an intelligible fashion and written in standard English?

PLOS ONE does not copyedit accepted manuscripts, so the language in submitted articles must be clear, correct, and unambiguous. Any typographical or grammatical errors should be corrected at revision, so please note any specific errors here.

Reviewer #1: Yes

Reviewer #2: Yes

**********

6. Review Comments to the Author

Please use the space provided to explain your answers to the questions above. You may also include additional comments for the author, including concerns about dual publication, research ethics, or publication ethics. (Please upload your review as an attachment if it exceeds 20,000 characters)

Reviewer #1: I am satisfied with the current version of the manuscript as authors adequately responded to all my comments.

Reviewer #2: (No Response)

**********

7. PLOS authors have the option to publish the peer review history of their article (what does this mean?). If published, this will include your full peer review and any attached files.

If you choose “no”, your identity will remain anonymous but your review may still be made public.

**Do you want your identity to be public for this peer review?** For information about this choice, including consent withdrawal, please see our Privacy Policy.

Reviewer #1: **Yes: **Deep Sharma

Reviewer #2: No

# Editor report: Acceptance letter

**DOI:** 10.1371/journal.pone.0246363.r004

**Authors:** Saikat Dewanjee

12 Feb 2021

PONE-D-20-24159R1 

Anti-oxidant activity and laxative effects of tannin-enriched extract of *Ecklonia cava* in loperamide-induced constipation of SD rats 

Dear Dr. Hwang:

I'm pleased to inform you that your manuscript has been deemed suitable for publication in PLOS ONE. Congratulations! Your manuscript is now with our production department. 

If your institution or institutions have a press office, please let them know about your upcoming paper now to help maximize its impact. If they'll be preparing press materials, please inform our press team within the next 48 hours. Your manuscript will remain under strict press embargo until 2 pm Eastern Time on the date of publication. For more information please contact onepress@plos.org.

If we can help with anything else, please email us at plosone@plos.org. 

Thank you for submitting your work to PLOS ONE and supporting open access. 

Kind regards, 

PLOS ONE Editorial Office Staff

on behalf of

Dr Saikat Dewanjee 

Academic Editor

PLOS ONE
